# Altered Retinoic Acid Metabolism in Diabetic Mouse Kidney Identified by ^18^O Isotopic Labeling and 2D Mass Spectrometry

**DOI:** 10.1371/journal.pone.0011095

**Published:** 2010-06-14

**Authors:** Jonathan M. Starkey, Yingxin Zhao, Rovshan G. Sadygov, Sigmund J. Haidacher, Wanda S. LeJeune, Nilay Dey, Bruce A. Luxon, Maureen A. Kane, Joseph L. Napoli, Larry Denner, Ronald G. Tilton

**Affiliations:** 1 Department of Internal Medicine, University of Texas Medical Branch, Galveston, Texas, United States of America; 2 Department of Biochemistry and Molecular Biology, University of Texas Medical Branch, Galveston, Texas, United States of America; 3 Department of Ophthalmology and Visual Sciences, University of Texas Medical Branch, Galveston, Texas, United States of America; 4 Stark Diabetes Center, University of Texas Medical Branch, Galveston, Texas, United States of America; 5 McCoy Diabetes Mass Spectrometry Research Laboratory, University of Texas Medical Branch, Galveston, Texas, United States of America; 6 Sealy Center for Molecular Medicine, University of Texas Medical Branch, Galveston, Texas, United States of America; 7 Institute for Translational Science Biomedical Informatics Program, University of Texas Medical Branch, Galveston, Texas, United States of America; 8 Department of Nutritional Science and Toxicology, University of California, Berkeley, California, United States of America; University of Minnesota, United States of America

## Abstract

**Background:**

Numerous metabolic pathways have been implicated in diabetes-induced renal injury, yet few studies have utilized unbiased systems biology approaches for mapping the interconnectivity of diabetes-dysregulated proteins that are involved. We utilized a global, quantitative, differential proteomic approach to identify a novel retinoic acid hub in renal cortical protein networks dysregulated by type 2 diabetes.

**Methodology/Principal Findings:**

Total proteins were extracted from renal cortex of control and *db/db* mice at 20 weeks of age (after 12 weeks of hyperglycemia in the diabetic mice). Following trypsinization, ^18^O- and ^16^O-labeled control and diabetic peptides, respectively, were pooled and separated by two dimensional liquid chromatography (strong cation exchange creating 60 fractions further separated by nano-HPLC), followed by peptide identification and quantification using mass spectrometry. Proteomic analysis identified 53 proteins with fold change ≥1.5 and p≤0.05 after Benjamini-Hochberg adjustment (out of 1,806 proteins identified), including alcohol dehydrogenase (ADH) and retinaldehyde dehydrogenase (RALDH1/ALDH1A1). Ingenuity Pathway Analysis identified altered retinoic acid as a key signaling hub that was altered in the diabetic renal cortical proteome. Western blotting and real-time PCR confirmed diabetes-induced upregulation of RALDH1, which was localized by immunofluorescence predominantly to the proximal tubule in the diabetic renal cortex, while PCR confirmed the downregulation of ADH identified with mass spectrometry. Despite increased renal cortical tissue levels of retinol and RALDH1 in *db/db* versus control mice, all-*trans*-retinoic acid was significantly decreased in association with a significant decrease in PPARβ/δ mRNA.

**Conclusions/Significance:**

Our results indicate that retinoic acid metabolism is significantly dysregulated in diabetic kidneys, and suggest that a shift in all-*trans*-retinoic acid metabolism is a novel feature in type 2 diabetic renal disease. Our observations provide novel insights into potential links between altered lipid metabolism and other gene networks controlled by retinoic acid in the diabetic kidney, and demonstrate the utility of using systems biology to gain new insights into diabetic nephropathy.

## Introduction

Diabetic nephropathy has become the most common cause of end-stage renal failure in the western world and its incidence continues to increase despite our understanding of the importance of microalbuminuria, glycemic control and blood pressure reduction. Many diabetic patients progress to end-stage renal disease despite recent advances in available therapeutics [1]. Numerous biochemical and metabolic pathways have emerged as predominant pathophysiological mechanisms for diabetes-induced renal injury [2]–[11]. The importance of each of these pathways is supported by a large volume of experimental animal data, and in some cases, clinical trials utilizing specific antagonism of a proposed biochemical mechanism. It is noteworthy that all of these proposed mechanisms involve dysregulated proteins. While there are probably numerous points of crosstalk between these different protein pathways, reductionist strategies are inherently unlikely to identify these interconnections. Only a systems biology approach can integrate the numerous proteins that are involved into higher-order cassettes operating within diabetic cells and tissues [12], [13], but few studies of diabetic complications have utilized this unbiased, global approach for mapping the interconnectivity of the different proteins postulated to have a causal role in diabetic nephropathy.

Using such an approach, we have identified previously unrecognized alterations in vitamin A metabolism as an important hub in protein networks dysregulated by diabetes in the renal cortex of type 2 diabetic mice. Vitamin A (retinol) functions in gene regulation for growth and development [14], [15]. These functions are regulated by numerous enzymes controlling a two step enzymatic reaction in which retinol is reversibly oxidized to bioactive retinaldehyde (retinal) followed by irreversible oxidation to the carboxylic acid, retinoic acid. Retinol is transported in the plasma via retinol binding protein (RBP), which essentially makes retinol available to all cells containing the intracellular lipid binding protein, cellular retinol binding protein (CRBP), which in turn, facilitates uptake of retinol into cells as well as its metabolism into retinyl esters or retinoic acid [16]. Elevated plasma levels of RBP4 have been implicated in the development of the metabolic syndrome and insulin resistance, and have been inversely correlated with adipocyte glucose transporter 4 (GLUT4) protein levels [17]. This metabolic pathway regulates gene expression via the carboxylic acid isomer, all-*trans*-retinoic acid (atRA) that is a ligand for two families of retinoid receptors – retinoic acid receptors (RAR) and retinoid X receptors (RXR). Although 9-*cis*-retinoic acid does serve as a pharmacological ligand for RXR and RAR, it has not been detected *in vivo* with rigorous analytical assays [18].

Despite a vast body of literature describing a role for retinoic acid as a critical regulator of gene expression modulating embryonic development and adult tissue regeneration, immune function, development, metabolism, and inflammation in multiple organ systems, little is known about the role of altered retinoic acid metabolism in diabetes, including diabetic nephropathy. Previously, changes in plasma levels of retinoids have been associated with type 1 diabetes, cardiovascular risk and cancer risk. A protective role of atRA has been shown in diabetic and nondiabetic proteinuric diseases [19], and a link between a cytochrome P450 enzyme (2E1) known to metabolize atRA and increased mitochondrial oxidative stress in type 1 diabetic rat kidneys has been established [20], [21]. Recently, atRA has been shown to bind PPARβ/δ and act as a ligand to activate transcription, suggesting that altered retinoic metabolism could provide a potential link to insulin resistance and fatty acid metabolism [22]. This has recently been demonstrated in mice fed high-fat diets [23]. Since others have reported that atRA failed to induce transcriptional activity of PPARβ/δ using reporter gene assays [24], this remains controversial. We report here that an enzyme involved in retinoic acid metabolism – RALDH1 (P24549; EC = 1.2.1.36) – is dysregulated in the renal cortex of 20 week-old *db/db* mice, a well characterized and widely used model of type 2 diabetes with consistent and relatively robust albuminuria (reflecting functional changes) and mesangial expansion, increased glomerular surface area, and tubulo-interstitial changes (reflecting early structural changes). Changes in RALDH1 are correlated with significant decreases in renal cortical levels of atRA and PPARβ/δ message despite elevated plasma levels of retinol and atRA. We also report that ADH1 (P00329; EC = 1.1.1.1) is dysregulated, although its role in physiological retinol metabolism remains controversial.

## Methods

### Animal and surgical protocols

Male Lepr^db^ (*db/db*) and age- and sex-matched control mice with the same genetic background (C57BLKS/J) were purchased from Jackson Labs (Bar Harbor, Maine) at 4 weeks of age, and were housed 2–3/cage in a sterile environment, in a room with a 12 hour light cycle and free access to standard chow and water. Body weight and nonfasting blood glucose were measured weekly. Mice were sacrificed 12 weeks after the onset of hyperglycemia, defined as a group mean value of 15 mmol/L, which occurred around 8 weeks of age. Age at the time of sacrifice was 20 weeks, representing a time point when albuminuria, mesangial expansion, increased glomerular surface area, and tubulo-interstitial changes are manifested [25]. In addition, significant renal cortical inflammation, reflecting induction of numerous NF-κB-regulated cytokines and chemokines, is present at this age [26]. Mice were anesthetized (ketamine/xylazine; 70/10 mg/kg i.p.), anticoagulated (5 units heparin), then exsanguinated prior to rapid aortic perfusion with ice-cold PBS to quickly rinse kidneys free of blood and to deliver protease and phosphatase inhibitors as described [26]. Both kidneys were removed, decapsulated, flash-frozen in liquid nitrogen, then stored at −80°C until processed. Prior to freezing, a 3–4 mm thick, coronal section through the middle of the kidney at the level of the renal pelvis was placed in 4% paraformaldehyde for 24 hours, and then transferred to Hanks Balanced Salt Solution (HBSS) for storage at 4°C until processed for immunohistochemistry.

### Ethics Statement

Animals were housed in the UTMB Animal Resource Center and used in accordance with its IACUC policies and the Public Health Service Guide for the Care and Use of Laboratory Animals.

### Tissue processing, protein extraction and tryptic digestion

Cortical tissue was separated from the medullary portion of each kidney under magnification with a dissecting microscope and suspended in 20 fold excess (wt/vol) of TRIzol® reagent (Invitrogen, Carlsbad, CA). The tissue was homogenized in a 1 ml Dounce homogenizer on ice. Proteins were extracted from the lysate according to the manufacturer's instructions, and the protein pellet was dissolved in 250 µl of 8 M guanidinium hydrochloride. In each experimental group, 300 µg of protein from each of five control mice or 5 diabetic mice were pooled, and the two pools containing 1.5 mg of protein were reduced with 10 mM dithiothreitol (DTT) for 30 min at room temperature. Protein cysteinyl residues were alkylated with 30 mM iodoacetamide for 2 h at 37°C. Each sample was diluted 10× with 100 mM ammonium bicarbonate, digested with 40 µg of trypsin overnight at 37°C, and each tryptic peptide mixture was desalted with a Sep-Pak® C18 cartridge (Waters, Milford, MA) following the manufacturer's instructions. Peptides were eluted from the cartridge with 80% acetonitrile and completely dried using a Speedvac.

### Post-proteolysis ^18^O-labeling

The ^18^O-labeling was performed as described [27] with slight modifications. The dried peptide samples were redissolved with 3 µl of anhydrous acetonitrile, 10 mg of immobilized trypsin (Applied Biosystems, CA) and 200 µl of normal water (H_2_^16^O) or H_2_^18^O containing 50 mM ammonium bicarbonate were added to the diabetic and control peptides, respectively, and both samples were incubated for 48 h at 37°C. Supernatants were collected using a spin column, and the corresponding ^18^O- and ^16^O-labeled samples were pooled and dried with a Speedvac. The ^18^O-/^16^O-labeled peptide mixture was desalted using a Sep-Pak®C18 cartridge (Waters Corp., Milford, MA) following the manufacturer's instructions; peptides were eluted from the cartridge with 80% acetonitrile and dried.

Dried peptide pools were redissolved by adding 20 µl of acetonitrile and diluted with 100 µl of 5 mM ammonium formate, pH 2.7. The pooled mixture was loaded onto a strong cation exchange (SCX) column (4.6 mm×25 cm; Poly LC, Columbia, MD) and separated with a linear gradient from 100% buffer A (5 mM ammonium formate-20% acetonitrile, pH 2.7) to 25% buffer B (1 M ammonium formate-20% acetonitrile, pH 3.0) over 40 min at a flow rate of 0.8 ml/min and followed by a linear gradient from 25% buffer B to 60% buffer B over 20 min. The eluate was manually collected after the first salt gradient was started. Sixty fractions, corresponding to 0.8 ml each, were collected and dried using a Speedvac.

### Two-dimensional liquid chromatography - tandem mass spectrometry (2D LC-MS/MS)

Each SCX fraction was redissolved in 20 µl of 0.1% trifluorocetic acid (TFA) and was injected onto a C18 peptide trap (Agilent, Santa Clara, CA), desalted with 0.2% formic acid at a flow rate of 10 µl/min for 180 min. Peptides were eluted from the trap and separated on a reversed phase nano-HPLC column (PicoFritTM, 75 µm×10 cm; tip ID 15 µm) with a linear gradient of 0–50% mobile phase B (0.1% formic acid - 90% acetonitrile) in mobile phase A (0.1% formic acid) over 120 min at 200 nl/min. LC-MS/MS experiments were performed with a LTQ linear ion trap mass spectrometer (ThermoFinnigan, San Jose, CA) equipped with a nanospray source; the mass spectrometer was coupled on-line to a ProteomX® nano-HPLC system (ThermoFinnigan, San Jose, CA). The mass spectrometer was operated in the data-dependent mode using Xcalibur software. The three most intense ions in each MS survey scan were automatically selected for Zoomscan and MS/MS.

### Data pre-processing

The acquired MS/MS spectra were searched with SEQUEST algorithm against a composite target-decoy mouse protein database consisting of the protein sequences (target) downloaded from SWISSPROT mouse protein database (downloaded July 2006) and reversed versions of these sequences (decoy) as described [28]. All SEQUEST searches were performed on the Bioworks 3.2 platform (ThermoFinnigan, San Jose, CA) using the following parameters: fully tryptic peptide (both tryptic terminus for all peptides), a mass tolerance of ±2.0 Da for precursor ion and ±1.0 Da for fragment ion. The output data from these searches were filtered and sorted by the DTASelect software [29] as previously described [28]. Only the top one peptide sequence match to each acquired MS/MS spectrum was considered. The criteria used in DTASelect were as follows: First, relatively conservative criteria (Sp≥300; ΔCn≥0.12; Xcorr of 1.9, 2.0 and 3.0 for data from a singly, doubly or triply charged precursor ions, respectively) were applied. Second, proteins that passed these thresholds were separated into two groups: proteins identified with two or more peptides and proteins identified with one peptide. Third, since the majority of the false positive identifications in our dataset were within the group of proteins with one identified peptide, much stricter criteria (Xcorr 2.2, 3.2, or 3.75 for precursor charge states of 1+, 2+, or 3+, respectively) were applied to the peptide hits in this group to increase identification certainty. Fourth, if multiple spectra were identified to match precisely the same sequence and charge state, only the spectrum with the highest Xcorr was retained. Finally, proteins that shared all matched peptides with other (homologous) proteins were removed. The false discovery rate (FDR) of identification was calculated to be less than 1% as described [30].

### Quantification of Differential Expression

The abundance ratios of ^18^O/^16^O-labeled peptide pairs were calculated with in-house software using the following equation [31]:
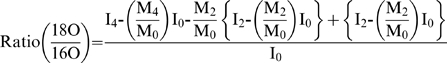
where *I_0_*, *I_2_* and *I_4_* are the measured relative peak intensities for the monoisotope peak for an unlabeled peptide, the peak with masses 2 Da higher, and the peak with 4 Da higher masses, respectively; *M_0_*, *M_2_*, and *M_4_* are the theoretical relative intensities for monoisotopic peak of the unlabeled peptide, the peaks with masses 2 Da and 4 Da higher than the monoisotopic peak, respectively. The “M values” were calculated based on the elemental composition of the peptide by using MS-isotope pattern calculator (http://prospector.ucsf.edu). If a peptide was identified more than once, a mean and standard deviation were calculated. The peptide ratios were averaged for all peptides for each protein to give a ratio per protein.

### Data and Statistical Analysis

^16^O/^18^O ratios that were ≤0 or ≥50 and UniProt identifications containing less than 5 individual peptide measurements were removed from further analysis. Calculated ratios were inverted (to generate a diabetes/control ratio) and log_2_ transformed prior to calculating mean, standard deviation and p-values by independent one-sample t-tests. Benjamini-Hochberg corrections for multiple testing comparisons were performed as indicated [32]. Data were analyzed through the use of Ingenuity Pathways Analysis (Ingenuity Systems®) with a fold change of ≥1.5 and p≤0.05 with Benjamini-Hochberg correction for multiple testing.

### Immunoblotting

Renal cortex (∼50 mg) was homogenized on ice using Wheaton 1 ml Dounce homogenizers in a 20-fold excess [wt/vol] of tissue extract buffer [50 mM HEPES (pH 7.0), 10 mM potassium chloride, 1 mM EDTA, 1 mM EGTA, protease inhibitor cocktail (Sigma P8340), phosphatase inhibitors (1 mM orthovanadate and 30 mM sodium fluoride), 1 mM DTT, and 0.5 mM PMSF]. Tissue lysates were electrophoresed on 10% SDS-polyacrylamide gels and analyzed by immunoblotting after transfer to nitrocellulose membranes (Bio-Rad Laboratories, Hercules, CA), using primary antibodies according to each manufacturer's instructions, followed by species-specific secondary antibodies tagged with a fluorescent dye (IR Dye™ 800; Rockland) at a 1∶5,000 dilution. Densitometric quantification of each protein was performed using the LI-COR Bioscience Odyssey™ Imaging System (LI-COR Bioscience, Lincoln, NE) as described [26]. β-actin was measured on every blot for evaluation of protein loading.

### Real Time RT-PCR

From each mouse, renal cortical tissue was suspended in 20 fold excess (wt/vol) of TRIzol® reagent (Invitrogen, Carlsbad, CA), homogenized in a 1 ml Dounce homogenizer on ice, and RNA extracted according to the manufacturer's instructions. cDNA was synthesized from 5 µg of mRNA using Superscript III (Invitrogen, Carlsbad, CA) according to the manufacturer's instructions. Relative quantification of select mRNA was performed on 1 µl of cDNA using a MyiQ Single-Color Real-Time PCR Detection System and iQ SYBR Green Supermix (Bio-Rad Laboratories, Hercules, CA) according to the manufacturer's instructions. All primers were purchased from SABiosciences, Inc (Frederick, MD). Data were analyzed using the ΔCT method in reference to cyclophilin (for RALDH1 and ADH) or GAPDH [33].

### Measurement of retinoids

Tissue and plasma samples were collected and handled under yellow lights. Tissue samples were homogenized by hand in ground glass homogenizers (Kontes, size 22) on ice in 1.0 to 2.0 ml saline (0.9% NaCl). Tissue and plasma samples were extracted as described [18], [34], [35]. Retinoic acid was quantified by LC/MS/MS with atmospheric pressure chemical ionization (APCI) in positive ion mode on an API-4000 (Applied Biosystems) as described [18], [34]. Retinol and retinyl ester were quantified by HPLC/UV on an Alliance 2690 (Waters) as described [35]. Tissue retinoids are expressed as mol/g tissue and plasma retinoids are expressed as mol/ml plasma.

### Immunofluorescent Imaging

Paraffin-embedded 5-µm coronal sections through the mid-line of the kidney at the level of the renal pelvis were washed in xylene twice for 5 min each, rehydrated by successive rinsing in 100, 95, 75, and 50% ethanol followed by phosphate-buffered saline (PBS), and antigen retrieval was performed using 10 mmol/l sodium citrate (pH 6.0) with 0.1% Triton X for 20 min at 95°C. Slides were blocked using nonimmune sera from the secondary antibody species diluted to 2% in PBS containing 0.2% cold fish skin gelatin, 0.1% saponin, and 0.05% Tween 20 for 2 h at room temperature. Primary antibody directed against RALDH1 (1∶200 dilution; Novus Biologicals) was incubated overnight at 4°C in blocking buffer containing 2% bovine serum albumin substituted for the nonimmune sera. Tissue sections were washed three times in PBS for 10 min each, and secondary antibody (labeled with IR Dye™ 680 for Li-COR imaging and AlexaFluor 633 (Molecular Probes) for confocal microscopy imaging) was added for 2 h at room temperature. Images were obtained on a Li-COR Bioscience Odyssey™ Imaging System (low magnification) and a Zeiss LSM 510 UV META Laser Scanning Confocal Microscope (high magnification). Negative controls included omitting the primary antibody.

### Statistical analysis

Statistical analysis of the proteomics data was detailed above. For all other data, the variance was reported as either standard deviations or standard errors of the mean, and indicated in the legends for each Table and Figure. Student's t-test with unequal variance was used to calculate significance of diabetic to control comparisons.

## Results

### Body weights and blood sugar data

Body weights of *db/db* mice were increased significantly at the onset of hyperglycemia (∼8 weeks of age), peaked by 12 weeks of age (∼2 fold increase over age-matched controls), then remained constant over the next two months (Table 1). After 3 months of hyperglycemia (20 weeks of age), the *db/db* mice exhibited significantly elevated non-fasted blood glucose levels. We have previously demonstrated persistent hypercholesterolemia, hyperinsulinemia, hyperleptinemia, and elevated plasma amylin levels in *db/db* vs control mice with this age and duration of hyperglycemia [26].

**Table 1:** Gravimetric and metabolic data for control and db/db mice.

Measurement	Control	*db/db*
Body weight (g)		
Initial	19±3	33±2 *
12 weeks	23±3	42±2 *
20 weeks	26±2	48±1 *
Blood glucose (mmol/l)		
Initial	9.2±2.1	19.3±1.3 *
12 weeks	7.9±1.2	27.6±3.4 *
20 weeks	7.9±1.1	29.4±3.0 *

Values expressed as means±SD. Controls, n = 10; *db/db*, n = 10.

Initial body weights correspond to the onset of hyperglycemia which occurs around 6–8 weeks of age.

Significantly different from controls by Students t-test: * p<0.0001.

### Diabetes-dysregulated renal cortical proteome

Each SCX fraction was submitted for mass spectrometry analysis three separate times, generating over 880,000 MS/MS spectra that were used for database searching with the SEQUEST algorithm. A total of 30,117 peptides were identified with ≤1% FDR and were mapped to 1,806 proteins, of which 796 proteins (44%) were identified with 5 or more peptides. Only these proteins were used for subsequent statistical and bioinformatics analyses. Table S1 lists the complete peptide data set for this experiment, while Table S2 lists the UniProt accession number, gene name, number of peptides used in the protein identification, mean fold change score, alpha value, and q* score (Benjamini-Hochberg adjusted p value) for the 796 proteins identified with 5 or more peptides.

### Identification of dysregulated vitamin A networks in the diabetic renal cortical proteome

**Figure 1:**
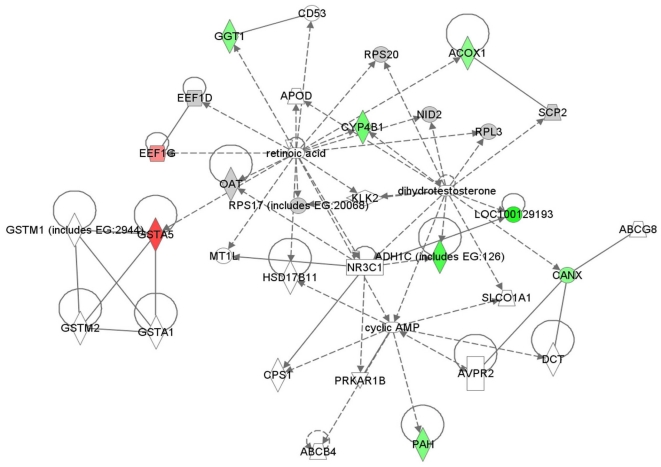
Retinoic acid is a key signaling hub in the highest ranked network generated by IPA. Protein-protein associations are indicated by edges containing single lines, whereas proteins that act upon another protein (controlling their expression) are indicated by arrows. Nodes are represented by shapes and colors: Shapes indicate function: enzymes (diamond), transcription regulators (oval), nuclear receptors (rectangle), cytokines (square), transporter (trapezoid), and “other” (circles). The red color indicates those proteins that are significantly increased in abundance in the diabetic kidney while green indicates those that are significantly decreased. The intensity of the color represents the degree of change. Abbreviations of proteins identified as significantly changed from the mass spectrometry dataset (indicated in green and red) are: GGT1, gamma-glutamyltransferase 1; ACOX1, acyl-Coenzyme A oxidase 1; CYP4B1, cytochrome P450, family 4, subfamily B, polypeptide 1; ADH1C, alcohol dehydrogenase 1C; CANX, calnexin; PAH, phenylalanine hydroxylase; EEF, elongation factor; GSTA5, glutathione S-transferase alpha 5.

After statistical analysis (fold change ≥1.5 and p≤0.05 after Benjamini-Hochberg adjustment) of the 796 proteins, 53 proteins were identified as significantly dysregulated in renal cortex (these proteins are highlighted in Table S2). The metabolite retinoic acid was identified as a key signaling hub in one of the highest ranked protein networks generated by Ingenuity Pathway Analysis (IPA) (score = 17) (Figure 1). Table 2 presents a list of the proteins identified as significantly altered in fatty acid (the highest ranked dysregulated protein network) and retinoic acid metabolism, and include alcohol dehydrogenase (ADH1; downregulated 2.5 fold), and retinaldehyde dehydrogenase 1 (ALDH1A1/RALDH1; upregulated 3 fold).

**Table 2:** Selected list of proteins significantly altered by diabetes involved in fatty acid and retinoic acid metabolism.

Gene symbol	UniProt Accession	Entrez gene name	Fold change	q*†
**ALDH1A1**	**P24549**	**aldehyde dehydrogenase 1 family, member A1**	**2.99**	**5.42E-03**
MCAT	Q8R3F5	malonyl CoA:ACP acyltransferase (mitochondrial)	2.09	1.02E-02
ACSL1	P41216	acyl-CoA synthetase long-chain family member 1	2.06	3.22E-02
ACOT1	O55137	acyl-CoA thioesterase 1	1.55	3.35E-02
CYB5A	P56395	cytochrome b5 type A (microsomal)	−1.54	4.42E-02
ACOX1	Q9R0H0	acyl-Coenzyme A oxidase 1, palmitoyl	−1.59	3.05E-06
ACSM1	Q91VA0	acyl-CoA synthetase medium-chain family member 1	−1.64	1.76E-03
APOA1	Q00623	apolipoprotein A–I	−1.67	1.67E-04
GGT1	Q60928	gamma-glutamyltransferase 1	−1.67	7.06E-04
CROT	Q9DC50	carnitine O-octanoyltransferase	−1.93	2.88E-07
ALDH3A2	P47740	aldehyde dehydrogenase 3 family, member A2	−2.05	2.85E-04
ACSM3	Q3UNX5	acyl-CoA synthetase medium-chain family member 3	−2.14	4.73E-03
UGT2B17	P17717	UDP glucuronosyltransferase 2 family, polypeptide B17	−2.23	5.19E-03
**ADH1C**	**P00329**	**Alcohol dehydrogenase 1C (class 1)**	**−2.49**	**5.11E-12**

Data are sorted by the fold change score. Two highlighted proteins represent key enzymes in retinol metabolism to retinoic acid.

† q* represents the False Discovery Rate.

### Alterations in Expression of Retinoid Metabolic Enzymes

**Figure 2:**
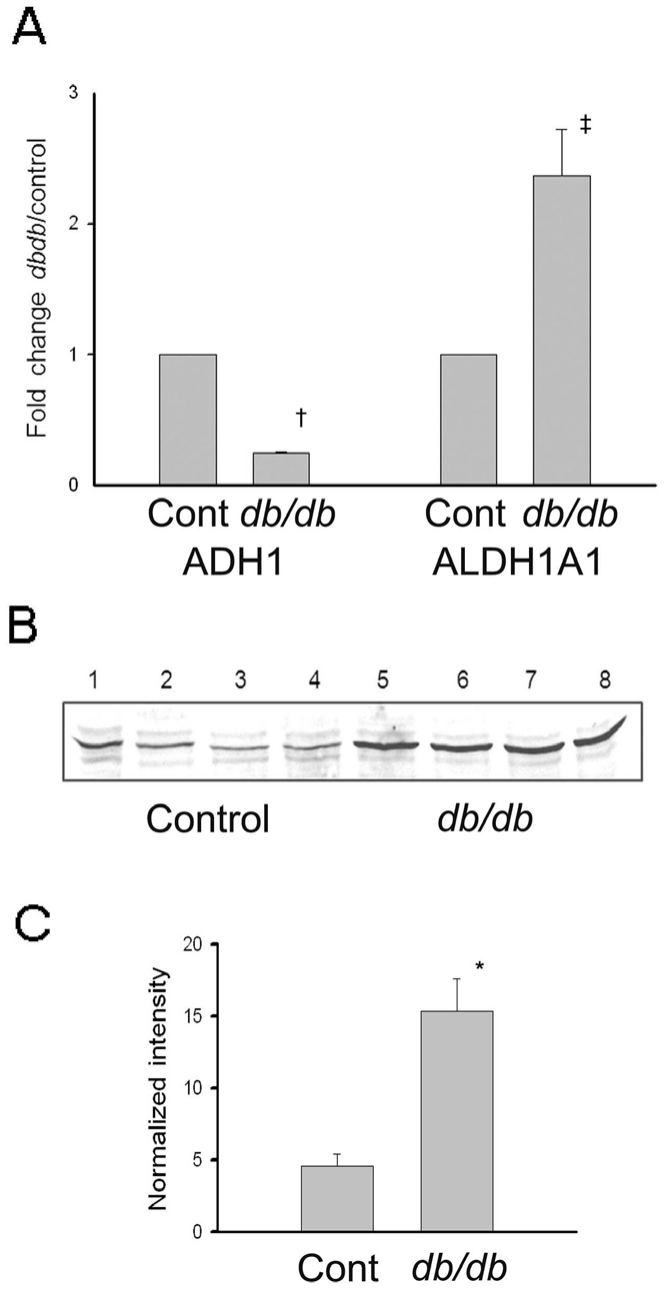
Real time-PCR and western blot of RALDH1 (ALDH1A1) and real time-PCR of ADH. Real time-PCR was analyzed using the ΔCT method and results are scaled to the mean control values ±SE (n = 7). Western blots were performed in duplicate and normalized to β-actin (n = 7) and results are represented as mean±SE. **A**. RNA was extracted from 50 mg of kidney cortical tissue and cDNA synthesis performed according to the manufacturer's instructions. Relative quantification of RALDH1 and ADH mRNA was performed using a MyiQ Single-Color Real-Time PCR Detection System. Control (n = 4) and diabetic (n = 7) results are normalized to the expression level of cyclophilin within each animal, and then plotted as fold change compared to the average control value. **B**. RALDH1 Western blots. Total tissue extracts were prepared from renal cortex of 3-month diabetic and age-matched control mouse kidneys. An equal amount of protein (50 µg/lane) was used for all animals, and representative control (lanes 1–4) and *db/db* (lanes 5–8) Western blots are shown. **C**. Densitometric quantification of RALDH1 for 7 control and 7 diabetic mice. Results are expressed as a mean and SD of the control and diabetic band intensities after normalization to β actin in each lane. Student's t-test: *p<0.005; ^†^p<0.001; ^‡^p<0.01.

**Figure 3:**
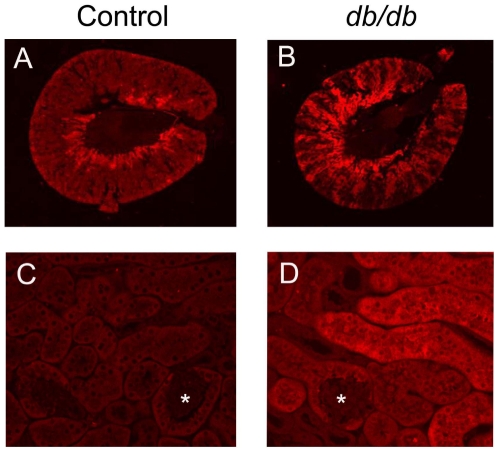
Immunohistochemical staining of RALDH1. Representative 5 µm formalin-fixed sections of kidney are shown from control (panels A and C) and *db/db* (panels B and D) mice. Panels A and B are low magnification images of the entire kidney section scanned using the LI-COR Bioscience Odyssey™ Imaging System while higher power confocal images are shown in Panels C and D. Negative controls included omitting the primary antibody, rabbit monoclonal anti-RALDH1 from Novus Biologicals (data not shown). Asterisks indicate glomeruli. Magnification for panels C and D, 300×.

In order to independently validate the mass spectrometry-derived fold change in expression for these two enzymes, we next used real time-PCR and immunoblotting to measure their expression levels. Both mRNA (Figure 2A) and protein (Figure 2B) levels of RALDH1 were increased significantly in *db/db* versus control renal cortex, while mRNA of ADH1 was decreased significantly (Figure 2A). These results confirmed the proteomics-based expression levels. Also, RALDH1 was upregulated by a small (1.64 fold) but significant (p<0.05) amount in the liver of *db/db* mice and in the renal cortex of 12 week old *db/db* mice (data not shown). Using the LI-COR imaging system, immunostaining for RALDH1 was located in the medullary portion of the control mouse kidney (Figure 3A) with lesser amounts present in the renal cortex near the surface of the kidney. In the *db/db* mouse kidney, increased staining for RALDH1 was evident in both renal medullary and cortical locations (Figure 3B). At higher magnification, renal cortical RALDH1 was predominantly located in tubular epithelium of *db/db* mice (Figure 3D) with little staining evident in diabetic glomeruli or in renal cortex sampled from controls (Figure 3C). A low magnification image of a coronal section through the mid-line of the kidney at the level of the renal pelvis is shown in Figure S1. This image is a composite of three Hematoxylin & Eosin stained images from the surface of the kidney to the outer portion of the renal medulla.

### Quantitative Measurement of Retinoids

**Figure 4:**
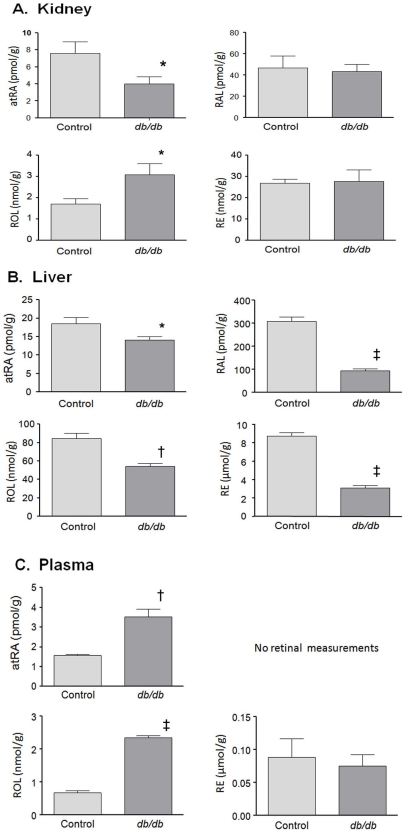
Quantification of retinoids extracted from tissue and plasma samples. Retinoids were quantified by LC/MS/MS as described [18], [34], while retinol and retinyl ester were quantified by HPLC/UV as described [35]. Tissue retinoids are expressed as mol/g tissue and plasma retinoids are expressed as mol/mL plasma. Student's t-test: *, p<0.05; ^†^p<0.005; ^‡^p<0.0001.

Since enzymes involved in retinoid metabolism were dysregulated in the *db/db* mouse kidney, we next measured retinoid levels in selected tissues and plasma. Retinol (ROL) levels were increased 3.5 fold in plasma (Figure 4C, lower left panel) and 1.8 fold in renal cortex (Figure 4A, lower left panel) of *db/db* vs control mice, but decreased 0.64 fold in liver (Figure 4B, lower left panel). While retinyl ester (RE) levels were unchanged in plasma and kidney, this metabolite was significantly decreased in the liver (0.4×control). Retinaldehyde (RAL) was not measured in plasma and unchanged in renal cortex, but was significantly decreased in diabetic liver (0.3×control). All-trans retinoic acid (atRA) was increased 2.3 fold in diabetic vs control plasma, but was significantly decreased in both liver and renal cortex compared to controls (0.76 and 0.53×, respectively). These results suggest a redistribution of all retinoid metabolites from liver into plasma and kidney, yet substantial decreases in atRA in renal cortex and liver despite elevated plasma levels suggest decreased synthesis and/or increased metabolism of atRA in these tissues.

### Assessment of atRA metabolizing enzymes

**Figure 5:**
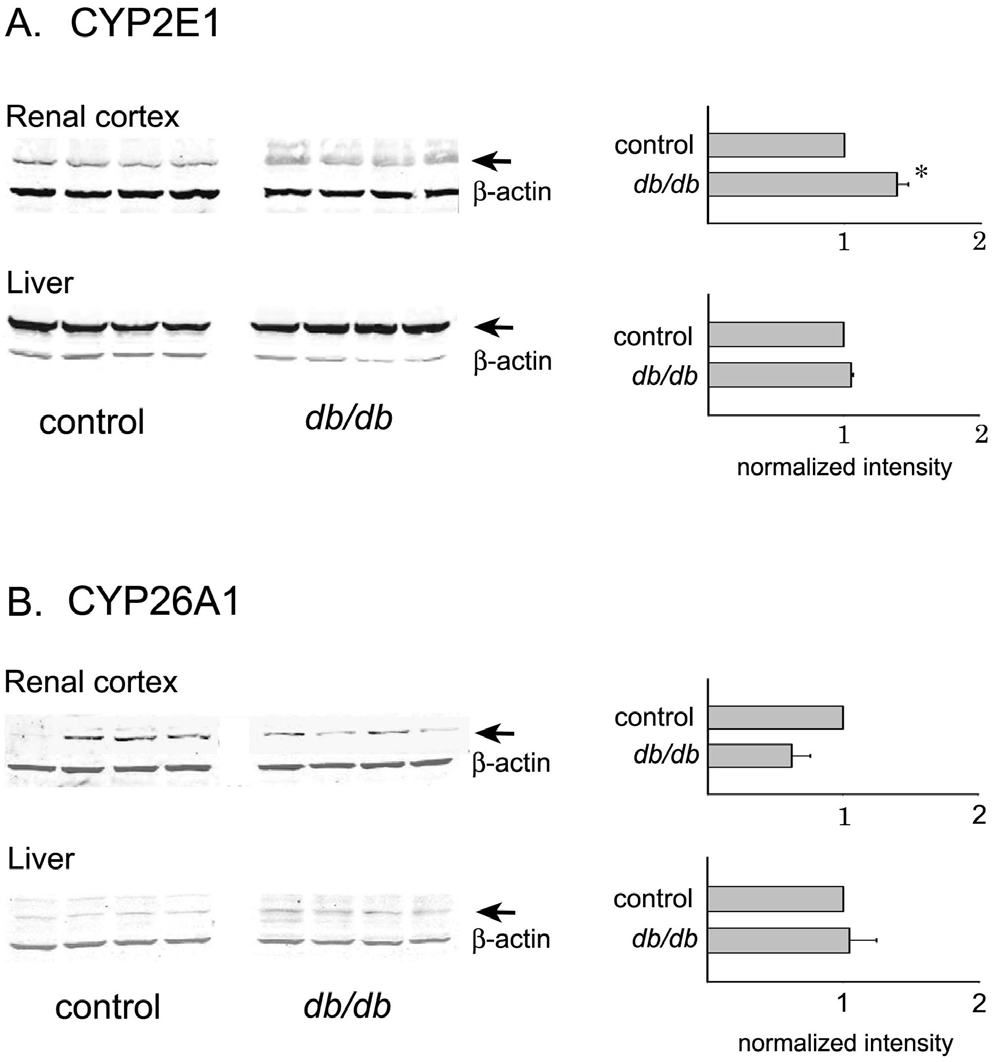
Western immunoblots of CYP2E1 and CYP26A1. Total tissue extracts were prepared from renal cortex and liver of 3-month diabetic and age-matched control mouse kidneys. An equal amount of protein (50 µg/lane) was used for all animals, and representative control (lanes 1–4) and *db/db* (lanes 5–8) tissue extracts are shown on the left. Densitometric quantitation of 7 control and 7 *db/db* kidneys and livers are shown on the right. Due to group differences in the expression level of β-actin caused by diabetes, results were not normalized to the loading control. Student's t-test: * p<0.02.

Since atRA levels were decreased despite increases in RALDH1 and unchanged levels of the intermediary substrate retinaldehyde, we next used immunoblotting to probe expression levels of several p450 enzymes previously reported to metabolize atRA, including cyp2E1, cyp26A1, and DHRS9 (NADP-dependent retinol dehydrogenase/reductase). Figure 5A demonstrates that cyp2E1 was weakly expressed in mouse renal cortex compared to its expression level in mouse liver, increased in diabetic kidney (p<0.02), and was unchanged in diabetic liver. CYP26A1 was weakly expressed in both kidney and liver, and while its expression was decreased slightly in the diabetic renal cortex, these differences were not significant due to significant heterogeneity within both control and diabetic animals. DHRS9 was undetectable in both kidney and liver using whole tissue extracts with the antibody used (Novus Biologicals; H00010170; data not shown).

### Real time-PCR of retinoid binding proteins, atRA-linked nuclear receptors, and target genes

**Figure 6:**
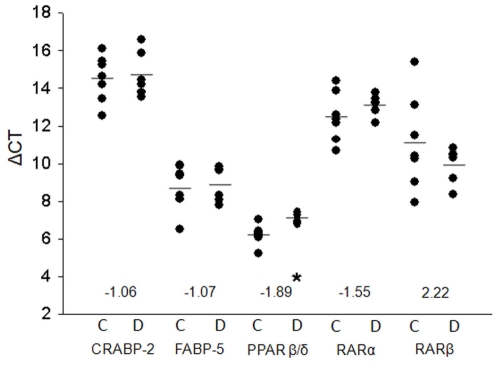
Real time-PCR of retinoid binding proteins and atRA-related nuclear receptors. Relative quantification of select mRNA was performed using a MyiQ Single-Color Real-Time PCR Detection System and iQ SYBR Green Supermix according to the manufacturer's instructions. All primers were purchased from SABiosciences, Inc. Data were analyzed using the ΔCT method in reference to cyclophillin or GAPDH. Controls, n = 7; *db/db*, n = 6. Significantly different from controls by Student's t-test: * p<0.005.

Recent reports have suggested that suppression of obesity and insulin resistance by atRA is largely mediated by PPARβ/δ [23], and this effect is enhanced by activation of retinoic acid receptors (RAR's), while others have reported that atRA does not compete with established PPARβ/δ agonists in ligand binding assays [24]. Since the potential interaction between atRA and PPARβ/δ remains controversial, we measured the mRNA expression level of this nuclear receptor as well as the retinoic acid receptor transcription factors and the relevant binding proteins. Figure 6 indicates that message levels for cytosolic retinoic acid binding protein (CRABP) and fatty acid binding protein (FABP) were unchanged by diabetes in the renal cortex, but that substantially more FABP message (mean ΔCT = 8.9) was present in the renal cortex than CRABP message (mean ΔCT = 14.5). PPARβ/δ also was abundant in the control renal cortex (mean ΔCT = 6.2) and was significantly decreased 1.9 fold in diabetic renal cortex (mean ΔCT = 7.1). While RARα was increased 1.6 fold, and RARβ decreased 2.2 fold, these changes were not significant due to substantial animal variability in the control mice.

## Discussion

We used a quantitative, stable isotope labeling approach combined with multidimensional LC-MS/MS to identify proteins differentially expressed in the renal cortex of the *db/db* mouse, a widely used model of type 2 diabetes. We then used IPA to map the significantly altered proteins identified with this experimental approach to protein pathways and networks dysregulated by diabetes, and bioinformatics to understand the relationships of these altered proteins in these pathways and networks. As a result, we identified a high ranking network containing the vitamin A metabolite retinoic acid as a significant signaling hub.

Retinoic acid is generated from retinol following the rate limiting step of retinaldehyde formation. Numerous dehydrogenases contributing to the metabolism of these retinoids have been reported, and generally fall into three major families: alcohol dehydrogenase (ADH) or short-chain dehydrogenase/reductase (SDR) enzymes that catalyze the reversible oxidation/reduction of retinol and retinaldehyde, and aldehyde dehydrogenases (ALDH) that catalyze the oxidation of retinaldehyde to retinoic acid [16], [36]. We identified ADH1 as being significantly downregulated in the renal cortex of *db/db* mice using quantitative mass spectrometry and supported this finding with real-time PCR. We also identified RALDH1 as being significantly upregulated, which was confirmed by real-time PCR, Western blotting, and immunohistochemistry. This finding is in agreement with a trend towards upregulation of RALDH1 in adipose tissue of *ob/ob* mice [37]. We also showed that RALDH1 was upregulated by a small but significant amount in the liver of *db/db* mice.

The role of ADH in the physiological generation of retinaldehyde remains controversial. Although some ADH convert retinol into retinaldehyde *in vitro*, these enzymes do not recognize the physiological form of retinol (holo-CRBPI) as substrate [38]. ADH1, and to a lesser extent ADH3, but not ADH4, convert high doses of retinol (50 mg/kg) into RA *in vivo* [39], [40], suggesting that pathological retinol doses can overwhelm homeostasis mechanisms and retinoid binding-protein capacities. Importantly, there are no reports demonstrating that overexpression of ADH enhances conversion of retinol into atRA, or that reduced ADH expression in cultured cells associates with a phenotype related to insufficient retinol activation. The observation in this study that retinaldehyde levels remained unchanged in the kidney despite significant decreases in ADH suggests that ADH is not responsible for the generation of retinaldehyde in the diabetic mouse kidney. The possibility that the microsomal SDRs may be the physiological converters of retinol to retinaldehyde in the kidney remains speculative at this time.

Despite >3 fold increases in plasma retinol levels and renal RALDH1 levels, we found that retinaldehyde was unchanged and atRA was significantly decreased in the diabetic renal cortex. The decrease in renal atRA may be due to decreased synthesis despite elevated RALDH1 levels and/or increased metabolism and clearance of retinoic acid. Alternatively, the decreased atRA may have caused a compensatory increase in RALDH1 since previous work has shown a negative feedback inhibition by atRA on the expression of RALDH1 [41]–[43]. Previous clinical and animal studies have focused primarily on plasma concentrations of retinol and found varying correlations in type 2 diabetes while demonstrating decreases in type 1 diabetes [44]–[48]. Our reported increase in plasma retinol in this model of type 2 diabetes is in contrast to the type 1 diabetes data, but is consistent with previous reports of diabetes-induced increases in RBP4, a retinol-binding protein which may be a biomarker of type 2 diabetes and insulin resistance [17]. Our finding that retinol, retinol esters, and retinaldehyde were significantly decreased in the liver suggests a redistribution of retinoids from liver and adipose stores (data not shown) accompanied by an increase in plasma retinoids in type 2 diabetes, and further suggests that simply measuring plasma retinol levels provides an incomplete understanding of altered vitamin A metabolism in diabetes. The implication(s) of this diabetes-induced redistribution of retinoids from liver, and their relevance to the progression of diabetic renal complications in this animal model, remain unknown at this time. However, these results are similar to previous studies of chronic ethanol intoxication where similar changes in retinoid distribution were reported to cause hepatic steatosis, fibrosis, and increased risk of hepatocellular carcinoma [49]. This also provides biological plausibility into the increased risk of hepatocellular carcinoma in patients with diabetes according to cohort studies [50]. Importantly, tissue atRA levels, despite varying plasma levels, are reduced in this animal model, suggesting that, at the site of retinoid function, there is the potential for reduced activity. Our observation of decreased tissue atRA despite increased plasma atRA is similar to the state of retinoic acid resistance seen in patients treated with atRA for promyelocytic leukemia. The retinoic acid resistance is believed to be caused by an increase in catabolism of atRA in tissues [51]. This observation may explain the failure of clinical trials utilizing carotenoids and/or retinoids for the prevention of cancer and cardiovascular disease [52], [53]. Despite evidence supporting the use of retinoic acid in the treatment of cancers, randomized controlled trials have not established efficacy and are not routinely used in clinical practice (with the notable exception of promyelocytic leukemia), and our data would suggest that the strategy of administering biologically inactive retinoids for prevention of disease as attempted in previous trials is unsuccessful because tissue metabolism dysregulation of atRA is causal for disease and not plasma and stored atRA precursors.

Given the redundancy in enzymes responsible for the generation and metabolism of retinoic acid, it is difficult to completely elucidate the pathophysiological mechanism responsible for the altered atRA levels in type 2 diabetes. However, our finding that diabetes increased renal cortical levels of CYP2E1 is similar to recent observations in streptozotocin-diabetic rats [20], [54], and offers a potential mechanism for increased metabolism of atRA. CYP2E1 is a classical ethanol-inducible P450 enzyme well known to metabolize fatty acids, lipid hydroperoxides, and ketone bodies [55] as well as atRA [49]. Importantly, it has been implicated in the generation of intramitochondrial reactive oxygen species [20], [56]. The diabetes-induced changes in this enzyme were significant but relatively small, and numerous other enzymes have been implicated in RA metabolism, including recent work suggesting that CYP3 subfamily may be involved in alterations of retinoic concentrations in non-retinoid toxicity states [57]. Nevertheless, similarities between ethanol-induced increased CYP2E1 and fatty liver damage and nonalcoholic-induced liver steatohepatitis in diabetes suggest common mechanisms involving CYP2E1 and atRA.

atRA exerts many of its biological activities by activating specific members of the nuclear hormone receptor superfamily of transcription factors. In addition to activating RAR, retinoic acid has been shown to be a ligand for the nuclear receptor PPARβ/δ [22], [58], although this remains controversial [24]. Recently, it was shown that there is a decrease in PPARβ/δ in adipose tissue of dietary obese mice [23]. Treatment with atRA restored this change, induced weight loss, and improved insulin responsiveness, suggesting that suppression of obesity and insulin resistance by atRA is largely mediated by PPARβ/δ. Similarly, we show decreases in PPARβ/δ mRNA expression and atRA in the kidney of *db/db* mice. The decrease in PPARβ/δ message is in contrast to PPARα, which we have shown to be increased in the kidney of *db/db* mice using proteomics techniques [59]. In that study, which used two dimensional gel electrophoresis and spot picking for mass spectrometry, we did not identify RALDH1 as significantly dysregulated and the importance of altered vitamin A metabolism was not appreciated. PPARα was identified as a significant hub in a high-ranking network generated by Ingenuity Pathways Analysis and confirmed by other investigators as significantly increased [60].

It previously has been reported that the partitioning of RAR and PPARβ/δ within cells is regulated by two members of the intracellular lipid-binding proteins – cellular RA-binding protein (CRABP) and one of the nine isotypes of fatty acid-binding protein (FABP-5). CRABP-2 shuttles RA to the RARs [61], while FABP-5 shuttles RA to PPARβ/δ [58], thus determining the spectrum of RA genes that are activated. While we did not demonstrate significant changes in either CRABP-2 or FABP-5 message in diabetic versus control kidneys, we did observe significantly more message for FABP-5 than CRABP-2. We reported significant decreases in atRA and in PPARβ/δ message, suggesting that genes activated by this signaling axis are impaired in the renal cortex of type 2 diabetes. Consistent with this suggestion was the observation that the PPARβ/δ-regulated protein, peroxisomal D3,D2-enoyl-CoA isomerase (PECI), was decreased in our proteomics dataset (−1.5 FC, BH<0.05) and confirmed by PCR (−1.9 FC, p<0.03) (data not shown). Our results are consistent with a recent report that renal expression of PPARβ/δ was significantly suppressed in animal models of type 1 diabetes [62]. In that study, it was hypothesized that reduced PPARβ/δ expression contributed to renal lipotoxicity secondary to reduced fatty acid oxidation. While we can associate changes in atRA levels with alterations in PPARβ/δ mRNA and most likely activity, we could not establish significant changes in the mRNA levels of FABP-5, CRABP-2, RARα, or RARβ. It therefore remains a question as to how the alterations in atRA levels modulate RAR activity. Regardless of the mechanism, atRA treatment is generally associated with a reduction of renal inflammation [19], [63], and therefore makes the use of retinoic acid metabolism blocking agents (RAMBA) a potential novel therapy for the treatment of diabetic nephropathy in which inflammation is a key contributor to the pathogenesis.

In conclusion, we have used a non-gel-based, ^18^O stable isotope labeling approach combined with 2D LC-MS/MS and bioinformatics to identify significant changes in protein expression, and to map these altered proteins to novel protein pathways and networks perturbed by diabetes in the renal cortex of the *db/db* mouse. This approach led to the discovery of decreased renal cortical levels of atRA in the diabetic mouse kidney. The pathophysiological mechanism(s) responsible for the decreased atRA remains to be elucidated at the cellular and molecular level. Likewise, potential causal links between decreased renal cortical atRA levels and progression of diabetic nephropathy and development of renal inflammation remain to be explored. Nevertheless, our observations provide novel insights into potential links between altered lipid metabolism and other gene networks controlled by retinoic acid in the diabetic kidney, and demonstrate the utility of using systems biology to gain new insights into diabetic nephropathy. The redistribution of retinoids observed in this model of type 2 diabetes suggests a new pathophysiological mechanism for which normalization of these changes may provide a novel therapy for both hepatic steatosis and diabetic renal complications.

## Supporting Information

Figure S1 Low magnification image of a renal coronal section. Paraffin-embedded 5-µm cross sections were obtained through the mid-line of the kidney at the level of the renal pelvis. This image is a composite of three Hematoxylin & Eosin stained images from the surface of the kidney to the outer portion of the renal medulla. 100×.
(2.18 MB EPS)

Table S1 Peptide Annotation Data
(6.92 MB XLS)

Table S2 Protein List
(0.18 MB PDF)
