# Supplementary material for: Altered Retinoic Acid Metabolism in Diabetic Mouse Kidney Identified by 18O Isotopic Labeling and 2D Mass Spectrometry
Source: PLoS One. 2010 Jun 14;5(6):e11095. doi: 10.1371/journal.pone.0011095 (PMC2885420; doi:10.1371/journal.pone.0011095)
Supplement: Table S2 — Protein List (0.18 MB PDF) [file pone.0011095.s003.pdf]

UniProt ID	Gene Name	Number of Peptides	Number of Unique Peptides	Mean Fold Change	alpha	q* (FDR)
O55060	THIOPURINE METHYLTRANSFERASE	11	4	-4.879	1.334E-02	6.515E-02
P11588	**MAJOR URINARY PROTEIN 1**	**32**	**2**	**-4.487**	**8.162E-20**	**2.166E-17**
Q8CH25	SAFB-LIKE, TRANSCRIPTION MODULATOR	7	1	-3.411	1.432E-02	6.785E-02
Q9JIX8	APOPTOTIC CHROMATIN CONDENSATION INDUCER 1	5	3	-3.101	2.102E-01	4.301E-01
P47941	V-CRK SARCOMA VIRUS CT10 ONCOGENE HOMOLOG (AVIAN)-LIKE	5	1	-2.818	6.219E-02	1.919E-01
P02088	**HEMOGLOBIN BETA CHAIN COMPLEX**	**36**	**3**	**-2.540**	**7.496E-13**	**4.590E-11**
P00329	**ALCOHOL DEHYDROGENASE 1 (CLASS I)**	**34**	**2**	**-2.487**	**7.058E-14**	**5.107E-12**
Q8BI84	MELANOMA INHIBITORY ACTIVITY 3	6	2	-2.341	1.024E-01	2.727E-01
Q61847	**MEPRIN 1 BETA**	**6**	**1**	**-2.278**	**2.205E-04**	**2.544E-03**
P11930	**NUDIX (NUCLEOSIDE DIPHOSPHATE LINKED MOIETY X)-TYPE MOTIF 19**	**20**	**4**	**-2.233**	**7.014E-05**	**9.626E-04**
P17717	**UDP GLUCURONOSYLTRANSFERASE 2 FAMILY, POLYPEPTIDE B5**	**8**	**1**	**-2.231**	**4.952E-04**	**5.186E-03**
Q9CZ44	SYNTAXIN BINDING PROTEIN 3A	11	4	-2.209	7.323E-02	2.167E-01
Q9R0M5	**THIAMIN PYROPHOSPHOKINASE**	**8**	**2**	**-2.154**	**2.684E-03**	**2.137E-02**
Q3UNX5	**ACYL-COA SYNTHETASE MEDIUM-CHAIN FAMILY MEMBER 3**	**18**	**3**	**-2.144**	**4.394E-04**	**4.726E-03**
Q61696	HEAT SHOCK PROTEIN 1B	11	4	-2.120	2.660E-02	1.075E-01
O35459	ENOYL COENZYME A HYDRATASE 1, PEROXISOMAL	5	1	-2.098	4.234E-01	6.371E-01
P47740	**ALDEHYDE DEHYDROGENASE FAMILY 3, SUBFAMILY A2**	**19**	**4**	**-2.054**	**1.466E-05**	**2.846E-04**
Q64462	**CYTOCHROME P450, FAMILY 4, SUBFAMILY B, POLYPEPTIDE 1**	**57**	**4**	**-2.024**	**8.181E-14**	**5.427E-12**
Q99K30	EPS8-LIKE 2	5	3	-1.998	1.387E-01	3.306E-01
Q91XE4	**ASPARTOACYLASE (AMINOACYLASE) 3**	**77**	**6**	**-1.938**	**1.841E-18**	**2.931E-16**
P51125	CALPASTATIN	5	3	-1.929	1.887E-01	3.984E-01
Q9DC50	**CARNITINE O-OCTANOYLTRANSFERASE**	**16**	**4**	**-1.927**	**8.317E-09**	**2.878E-07**
Q7TNG8	**LACTATE DEHYDROGENASE D**	**56**	**10**	**-1.910**	**9.198E-13**	**5.230E-11**
Q8JZZ0	**EXPRESSED SEQUENCE AI313915**	**16**	**3**	**-1.905**	**8.103E-12**	**4.031E-10**
Q62318	TRIPARTITE MOTIF PROTEIN 28	5	2	-1.896	2.093E-01	4.294E-01
P62874	GUANINE NUCLEOTIDE BINDING PROTEIN, BETA 1	8	2	-1.841	2.949E-02	1.168E-01
Q9DCQ2	**RIKEN CDNA 0610012D14 GENE**	**5**	**1**	**-1.835**	**4.152E-03**	**2.899E-02**
Q9R022	J DOMAIN PROTEIN 1	7	2	-1.830	8.304E-02	2.344E-01
P16331	**PHENYLALANINE HYDROXYLASE**	**26**	**7**	**-1.793**	**1.252E-03**	**1.120E-02**
Q61789	LAMININ, ALPHA 3	5	1	-1.782	1.437E-02	6.767E-02
Q9EPL9	ACYL-COENZYME A OXIDASE 3, PRISTANOYL	13	5	-1.766	9.447E-03	5.116E-02
Q8CFZ5	SOLUTE CARRIER FAMILY 22 (ORGANIC ANION/CATION TRANSPORTER), MEMBER 12	6	2	-1.744	6.025E-02	1.888E-01
P62918	RIBOSOMAL PROTEIN L8	8	1	-1.733	8.321E-02	2.341E-01
Q8BUV3	GEPHYRIN	5	1	-1.712	1.729E-01	3.751E-01
Q9DBL1	ACYL-COENZYME A DEHYDROGENASE, SHORT/BRANCHED CHAIN	5	1	-1.711	1.282E-01	3.160E-01
P52196	THIOSULFATE SULFURTRANSFERASE, MITOCHONDRIAL	15	5	-1.709	6.215E-02	1.925E-01
Q91Z53	GLYOXYLATE REDUCTASE/HYDROXYPYRUVATE REDUCTASE	15	3	-1.705	3.005E-02	1.179E-01
Q99J99	**MERCAPTOPYRUVATE SULFURTRANSFERASE**	**36**	**3**	**-1.692**	**8.186E-04**	**8.045E-03**

Q60928	**GAMMA-GLUTAMYLTRANSFERASE 1**	**35**	**8**	**-1.673**	**4.704E-05**	**7.064E-04**
P07724	**ALBUMIN 1**	**154**	**15**	**-1.671**	**5.774E-14**	**4.596E-12**
Q00623	**APOLIPOPROTEIN A-I**	**11**	**5**	**-1.667**	**7.957E-06**	**1.667E-04**
Q80XN0	**3-HYDROXYBUTYRATE DEHYDROGENASE, TYPE 1**	**8**	**1**	**-1.664**	**7.559E-03**	**4.392E-02**
Q8C0I1	ALKYLGLYCERONE PHOSPHATE SYNTHASE	17	4	-1.659	1.524E-01	3.475E-01
Q5XJY5	ARCHAIN 1	5	1	-1.643	5.655E-02	1.808E-01
Q91VA0	**ACYL-COA SYNTHETASE MEDIUM-CHAIN FAMILY MEMBER 1**	**54**	**9**	**-1.637**	**1.438E-04**	**1.761E-03**
P61358	RIBOSOMAL PROTEIN L27	7	2	-1.631	1.383E-01	3.326E-01
Q9CQC6	BASIC LEUCINE ZIPPER AND W2 DOMAINS 1	5	1	-1.628	3.195E-01	5.376E-01
Q9JIF7	COATOMER PROTEIN COMPLEX, SUBUNIT BETA 1	6	2	-1.621	3.836E-01	6.083E-01
Q8BVI4	DNA SEGMENT, CHR 5, ERATO DOI 371, EXPRESSED	11	2	-1.613	1.690E-01	3.717E-01
P35564	**CALNEXIN**	**32**	**3**	**-1.606**	**2.460E-04**	**2.758E-03**
Q9WU78	PROGRAMMED CELL DEATH 6 INTERACTING PROTEIN	5	3	-1.600	5.705E-02	1.817E-01
Q76MZ3	PROTEIN PHOSPHATASE 2 , REGULATORY SUBUNIT A (PR 65), ALPHA ISOFORM	9	4	-1.597	2.179E-01	4.392E-01
Q93092	TRANSALDOLASE 1	8	2	-1.595	2.913E-01	5.188E-01
Q61391	MEMBRANE METALLO ENDOPEPTIDASE	5	2	-1.592	1.510E-02	7.069E-02
P28825	MEPRIN 1 ALPHA	6	1	-1.590	1.569E-02	7.304E-02
P51410	RIBOSOMAL PROTEIN L9	10	3	-1.588	6.210E-02	1.931E-01
Q9R0H0	**ACYL-COENZYME A OXIDASE 1, PALMITOYL**	**40**	**8**	**-1.586**	**9.966E-08**	**3.051E-06**
Q8VEK0	**TRANSMEMBRANE PROTEIN 30A**	**7**	**1**	**-1.582**	**2.313E-04**	**2.631E-03**
P47753	CAPPING PROTEIN (ACTIN FILAMENT) MUSCLE Z-LINE, ALPHA 1	7	2	-1.575	1.225E-02	6.056E-02
P07758	SERINE (OR CYSTEINE) PEPTIDASE INHIBITOR, CLADE A, MEMBER 1A	11	2	-1.570	2.790E-02	1.122E-01
Q8JZN5	ACYL-COENZYME A DEHYDROGENASE FAMILY, MEMBER 9	12	3	-1.554	1.386E-01	3.314E-01
Q91WU5	ARSENIC (+3 OXIDATION STATE) METHYLTRANSFERASE	7	1	-1.544	4.838E-02	1.625E-01
P56395	**RIKEN CDNA 0610009N12 GENE**	**16**	**3**	**-1.541**	**7.664E-03**	**4.421E-02**
Q9D0J8	RIKEN CDNA 2610009E16 GENE	5	1	-1.540	3.501E-02	1.296E-01
P35293	**RAB18, MEMBER RAS ONCOGENE FAMILY**	**8**	**2**	**-1.539**	**3.766E-05**	**6.117E-04**
Q8R1Q8	DYNEIN CYTOPLASMIC 1 LIGHT INTERMEDIATE CHAIN 1	7	2	-1.538	3.129E-01	5.322E-01
Q9D6Y7	RIKEN CDNA 2310045J23 GENE	18	1	-1.535	1.431E-01	3.310E-01
P97447	FOUR AND A HALF LIM DOMAINS 1	6	1	-1.506	4.197E-02	1.465E-01
P06801	MALIC ENZYME, SUPERNATANT	11	3	-1.499	7.119E-02	2.122E-01
Q9DBM2	ENOYL-COENZYME A, HYDRATASE/3-HYDROXYACYL COENZYME A DEHYDROGENASE	57	13	-1.495	6.879E-06	1.521E-04
Q9QXY6	EH-DOMAIN CONTAINING 3	12	2	-1.494	6.699E-02	2.027E-01
P30416	FK506 BINDING PROTEIN 4	10	3	-1.487	1.021E-05	2.083E-04
Q99KB8	HYDROXYACYL GLUTATHIONE HYDROLASE	17	2	-1.480	1.299E-01	3.183E-01
P32848	PARVALBUMIN	24	2	-1.476	2.818E-03	2.178E-02
P01942	HEMOGLOBIN ALPHA, ADULT CHAIN 1	180	4	-1.475	5.748E-09	2.080E-07
P98078	DISABLED HOMOLOG 2 (DROSOPHILA)	34	6	-1.474	6.498E-03	3.918E-02
O35488	SOLUTE CARRIER FAMILY 27 (FATTY ACID TRANSPORTER), MEMBER 2	56	8	-1.473	9.274E-17	1.055E-14
P48678	LAMIN A	13	6	-1.471	1.708E-01	3.746E-01
P80318	CHAPERONIN SUBUNIT 3 (GAMMA)	10	6	-1.471	3.025E-01	5.245E-01

Q02248	CATENIN (CADHERIN ASSOCIATED PROTEIN), BETA 1	7	1	-1.469	4.105E-01	6.308E-01
Q6PB66	LEUCINE-RICH PPR-MOTIF CONTAINING	7	2	-1.469	1.236E-03	1.118E-02
P27773	PROTEIN DISULFIDE ISOMERASE ASSOCIATED 3	152	12	-1.468	2.436E-15	2.423E-13
Q8CGC7	GLUTAMYL-PROLYL-TRNA SYNTHETASE	6	2	-1.467	1.725E-01	3.752E-01
P48774	GLUTATHIONE S-TRANSFERASE, MU 5	8	3	-1.454	4.314E-01	6.407E-01
Q61316	HEAT SHOCK PROTEIN, 110 KDA	13	4	-1.453	9.840E-02	2.673E-01
Q3UMF0	COBL-LIKE 1	15	6	-1.449	1.193E-01	2.967E-01
Q3TNA1	XYLULOKINASE HOMOLOG (H. INFLUENZAE)	30	3	-1.448	1.156E-04	1.461E-03
O88569	HETEROGENEOUS NUCLEAR RIBONUCLEOPROTEIN A2/B1	84	6	-1.444	2.573E-06	6.400E-05
Q8BLF1	ARYLACETAMIDE DEACETYLASE-LIKE 1	8	2	-1.437	4.539E-02	1.564E-01
Q9CPY7	LEUCINE AMINOPEPTIDASE 3	60	14	-1.433	2.263E-11	1.001E-09
Q9QUM9	PROTEASOME (PROSOME, MACROPAIN) SUBUNIT, ALPHA TYPE 6	6	1	-1.432	2.897E-02	1.153E-01
Q99LF4	DNA SEGMENT, CHR 10, WAYNE STATE UNIVERSITY 52, EXPRESSED	8	1	-1.430	5.239E-03	3.390E-02
P48962	SOLUTE CARRIER FAMILY 25 (MITOCHONDRIAL CARRIER, ADENINE NUCLEOTIDE TRANSLOCATOR), MEMBER 4	17	2	-1.428	2.481E-01	4.747E-01
P17710	HEXOKINASE 1	5	3	-1.425	2.449E-01	4.721E-01
P24270	CATALASE	45	9	-1.422	1.090E-02	5.560E-02
Q91VI7	EXPRESSED SEQUENCE AW546468	13	6	-1.420	1.042E-01	2.712E-01
P40936	INDOLETHYLAMINE N-METHYLTRANSFERASE	23	4	-1.414	2.564E-02	1.047E-01
P35980	RIBOSOMAL PROTEIN L18	6	2	-1.414	4.280E-01	6.404E-01
P11862	GROWTH ARREST SPECIFIC 2	9	1	-1.411	3.905E-03	2.775E-02
Q9D826	PIPECOLIC ACID OXIDASE	8	2	-1.407	1.334E-02	6.476E-02
O55222	INTEGRIN LINKED KINASE	8	3	-1.405	4.246E-01	6.377E-01
P10852	SOLUTE CARRIER FAMILY 3 (ACTIVATORS OF DIBASIC AND NEUTRAL AMINO ACID TRANSPORT), MEMBER 2	7	3	-1.401	1.718E-01	3.748E-01
P07901	HEAT SHOCK PROTEIN 1, ALPHA	163	10	-1.400	1.892E-12	1.004E-10
P62245	RIBOSOMAL PROTEIN S15A	5	1	-1.397	2.872E-02	1.149E-01
Q7M6Y3	PHOSPHATIDYLINOSITOL BINDING CLATHRIN ASSEMBLY PROTEIN	12	3	-1.395	1.391E-01	3.285E-01
P57776	EUKARYOTIC TRANSLATION ELONGATION FACTOR 1 DELTA (GUANINE NUCLEOTIDE EXCHANGE PROTEIN)	18	4	-1.392	1.116E-01	2.838E-01
Q9WVK4	EH-DOMAIN CONTAINING 1	20	7	-1.388	2.350E-02	9.896E-02
Q01730	RAS SUPPRESSOR PROTEIN 1	5	2	-1.387	3.593E-01	5.825E-01
Q9ERU9	RAN BINDING PROTEIN 2	9	3	-1.386	2.302E-01	4.535E-01
Q60864	STRESS-INDUCED PHOSPHOPROTEIN 1	11	2	-1.381	5.621E-05	8.135E-04
Q9QXN5	MYO-INOSITOL OXYGENASE	12	3	-1.378	1.675E-02	7.619E-02
Q01768	EXPRESSED IN NON-METASTATIC CELLS 2, PROTEIN	33	2	-1.377	2.472E-05	4.186E-04
P19324	SERINE (OR CYSTEINE) PEPTIDASE INHIBITOR, CLADE H, MEMBER 1	6	2	-1.374	4.026E-02	1.424E-01
P42932	CHAPERONIN SUBUNIT 8 (THETA)	12	3	-1.368	8.434E-02	2.364E-01
P32020	STEROL CARRIER PROTEIN 2, LIVER	33	5	-1.362	4.314E-06	9.811E-05
Q62425	NADH DEHYDROGENASE (UBIQUINONE) 1 ALPHA SUBCOMPLEX, 4	11	1	-1.362	1.762E-01	3.802E-01
P14211	CALRETICULIN	44	7	-1.359	5.351E-08	1.704E-06

P17751	TRIOSEPHOSPHATE ISOMERASE 1	75	6	-1.356	9.961E-04	9.328E-03
Q9Z2W0	ASPARTYL AMINOPEPTIDASE	6	3	-1.350	1.192E-02	5.932E-02
Q9EQP2	EH-DOMAIN CONTAINING 4	18	8	-1.350	3.974E-02	1.412E-01
P61022	RIKEN CDNA 1500003O03 GENE	5	1	-1.348	2.981E-01	5.226E-01
Q8BFR5	TU TRANSLATION ELONGATION FACTOR, MITOCHONDRIAL	23	8	-1.348	2.649E-01	4.915E-01
P57759	ENDOPLASMIC RETICULUM PROTEIN 29	13	3	-1.347	8.773E-02	2.416E-01
P45376	ALDO-KETO REDUCTASE FAMILY 1, MEMBER B3 (ALDOSE REDUCTASE)	7	2	-1.340	7.098E-03	4.216E-02
P48036	ANNEXIN A5	12	1	-1.336	2.161E-01	4.378E-01
P70441	SOLUTE CARRIER FAMILY 9 (SODIUM/HYDROGEN EXCHANGER), ISOFORM 3 REGULATOR 1	26	4	-1.336	6.459E-02	1.978E-01
O35737	HNRNP H	9	1	-1.334	3.948E-02	1.415E-01
P53994	RAB2, MEMBER RAS ONCOGENE FAMILY	6	3	-1.334	5.178E-04	5.353E-03
P06797	CATHEPSIN L	6	1	-1.324	6.313E-01	8.027E-01
P35979	RIBOSOMAL PROTEIN L12	26	1	-1.323	8.523E-02	2.372E-01
Q60930	VOLTAGE-DEPENDENT ANION CHANNEL 2	38	7	-1.322	1.666E-03	1.426E-02
P08003	PROTEIN DISULFIDE ISOMERASE ASSOCIATED 4	19	6	-1.321	5.636E-03	3.589E-02
Q8BIJ6	RIKEN CDNA 2010002H18 GENE	5	1	-1.319	8.692E-03	4.838E-02
P18572	BASIGIN	7	2	-1.316	3.327E-01	5.552E-01
Q9D1A2	CNDP DIPEPTIDASE 2 (METALLOPEPTIDASE M20 FAMILY)	51	8	-1.314	1.349E-11	6.318E-10
P10637	MICROTUBULE-ASSOCIATED PROTEIN TAU	11	4	-1.313	1.716E-01	3.753E-01
Q61171	PEROXIREDOXIN 2	14	2	-1.310	3.953E-05	6.169E-04
Q62167	DEAD/H (ASP-GLU-ALA-ASP/HIS) BOX POLYPEPTIDE 3, X-LINKED	11	4	-1.308	4.635E-02	1.583E-01
Q8R311	MENINGIOMA EXPRESSED ANTIGEN 6 (COILED-COIL PROLINE-RICH)	5	1	-1.306	1.613E-03	1.396E-02
Q9DCG6	RIKEN CDNA 0610038K03 GENE	43	3	-1.303	1.383E-02	6.631E-02
Q8VCF0	RIKEN CDNA D430028G21 GENE	8	2	-1.301	2.788E-01	5.044E-01
O08795	PROTEIN KINASE C SUBSTRATE 80K-H	6	2	-1.298	3.569E-02	1.309E-01
P61161	ARP2 ACTIN-RELATED PROTEIN 2 HOMOLOG (YEAST)	6	2	-1.297	2.668E-01	4.928E-01
Q9DCY0	KIDNEY EXPRESSED GENE 1	6	2	-1.296	4.414E-01	6.483E-01
P63037	DNAJ (HSP40) HOMOLOG, SUBFAMILY A, MEMBER 1	7	1	-1.295	3.967E-02	1.416E-01
Q8K0L3	ACYL-COA SYNTHETASE MEDIUM-CHAIN FAMILY MEMBER 2	723	19	-1.285	3.178E-34	1.265E-31
Q8BHN3	ALPHA GLUCOSIDASE 2 ALPHA NEUTRAL SUBUNIT	6	1	-1.285	1.799E-02	8.138E-02
P10126	EUKARYOTIC TRANSLATION ELONGATION FACTOR 1 ALPHA 1	56	6	-1.283	3.823E-02	1.377E-01
O55125	4-NITROPHENYLPHOSPHATASE DOMAIN AND NON-NEURONAL SNAP25-LIKE PROTEIN HOMOLOG 1 (C. ELEGANS)	22	2	-1.283	2.282E-01	4.553E-01
O35381	ACIDIC (LEUCINE-RICH) NUCLEAR PHOSPHOPROTEIN 32 FAMILY, MEMBER A	6	1	-1.282	8.798E-03	4.863E-02
Q9EQU5	SET TRANSLOCATION	6	2	-1.279	3.483E-01	5.670E-01
Q60648	GM2 GANGLIOSIDE ACTIVATOR PROTEIN	14	1	-1.277	7.947E-06	1.710E-04
Q8R0Y6	RIKEN CDNA 1810048F20 GENE	51	15	-1.275	1.639E-02	7.542E-02
Q99P72	RETICULON 4	5	2	-1.275	3.803E-01	6.054E-01
Q9JHI5	ISOVALERYL COENZYME A DEHYDROGENASE	5	1	-1.271	1.138E-01	2.858E-01
Q8VCT4	CARBOXYLESTERASE 3	18	4	-1.271	2.157E-05	3.815E-04

Q9NYQ2	HYDROXYACID OXIDASE (GLYCOLATE OXIDASE) 3	22	5	-1.271	7.753E-02	2.228E-01
Q78JT3	3-HYDROXYANTHRANILATE 3,4-DIOXYGENASE	11	1	-1.271	5.668E-05	8.056E-04
O09174	2-METHYLACYL-COA RACEMASE	13	6	-1.271	8.291E-02	2.349E-01
Q9D7N9	RIKEN CDNA 2310001A20 GENE	7	1	-1.268	2.389E-01	4.649E-01
P12367	PROTEIN KINASE, CAMP DEPENDENT REGULATORY, TYPE II ALPHA	7	1	-1.267	1.018E-02	5.295E-02
Q64521	GLYCEROL PHOSPHATE DEHYDROGENASE 2, MITOCHONDRIAL	12	7	-1.260	1.408E-01	3.297E-01
P34914	EPOXIDE HYDROLASE 2, CYTOPLASMIC	44	11	-1.259	4.873E-02	1.630E-01
P61089	UBIQUITIN-CONJUGATING ENZYME E2N	6	1	-1.259	2.172E-01	4.388E-01
O08553	DIHYDROPYRIMIDINASE-LIKE 2	8	4	-1.255	5.311E-01	7.288E-01
P97450	ATP SYNTHASE, H+ TRANSPORTING, MITOCHONDRIAL F0 COMPLEX, SUBUNIT F	12	1	-1.251	2.085E-01	4.312E-01
P35505	FUMARYLACETOACETATE HYDROLASE	127	5	-1.251	5.863E-07	1.667E-05
Q8K370	ACYL-COENZYME A DEHYDROGENASE FAMILY, MEMBER 10	15	6	-1.250	2.491E-02	1.022E-01
P53026	RIBOSOMAL PROTEIN L10A	8	3	-1.245	1.100E-03	1.018E-02
P27612	PHOSPHOLIPASE A2, ACTIVATING PROTEIN	5	1	-1.244	7.323E-03	4.318E-02
P37040	P450 (CYTOCHROME) OXIDOREDUCTASE	10	3	-1.239	5.071E-01	7.044E-01
P24369	PEPTIDYLPROLYL ISOMERASE B	17	4	-1.239	5.259E-03	3.376E-02
Q99MR8	METHYLCROTONOYL-COENZYME A CARBOXYLASE 1 (ALPHA)	29	8	-1.238	2.929E-01	5.193E-01
Q9CRB9	COILED-COIL-HELIX-COILED-COIL-HELIX DOMAIN CONTAINING 3	7	2	-1.236	5.321E-01	7.289E-01
P62082	RIBOSOMAL PROTEIN S7	8	1	-1.235	4.895E-02	1.630E-01
Q8K0D5	G ELONGATION FACTOR 1	5	2	-1.235	2.460E-02	1.015E-01
P63276	RIBOSOMAL PROTEIN S17	9	1	-1.234	2.457E-01	4.724E-01
P56391	RIKEN CDNA 2010000G05 GENE	41	3	-1.227	9.594E-03	5.125E-02
P51881	SOLUTE CARRIER FAMILY 25 (MITOCHONDRIAL CARRIER, ADENINE NUCLEOTIDE TRANSLOCATOR), MEMBER 5	32	2	-1.222	6.078E-02	1.897E-01
Q02819	NUCLEOBINDIN 1	13	2	-1.217	5.995E-03	3.728E-02
Q02053	UBIQUITIN-ACTIVATING ENZYME E1, CHR X	27	5	-1.216	3.330E-02	1.262E-01
Q60759	GLUTARYL-COENZYME A DEHYDROGENASE	18	4	-1.216	2.964E-01	5.231E-01
Q91YJ2	SORTING NEXIN 4	13	1	-1.213	2.116E-01	4.318E-01
Q99LB2	DEHYDROGENASE/REDUCTASE (SDR FAMILY) MEMBER 4	6	2	-1.213	1.383E-01	3.317E-01
P08228	SUPEROXIDE DISMUTASE 1, SOLUBLE	32	3	-1.211	1.034E-02	5.347E-02
Q91V41	RAB14, MEMBER RAS ONCOGENE FAMILY	5	2	-1.210	6.683E-01	8.235E-01
P35700	PEROXIREDOXIN 1	15	6	-1.210	8.849E-03	4.858E-02
O88338	CADHERIN 16	49	9	-1.209	8.596E-02	2.376E-01
P11499	HEAT SHOCK PROTEIN 1, BETA	39	8	-1.209	2.094E-02	9.110E-02
P60335	POLY(RC) BINDING PROTEIN 1	9	3	-1.209	3.255E-01	5.443E-01
P56565	S100 CALCIUM BINDING PROTEIN A1	36	1	-1.205	6.321E-05	8.827E-04
P09055	INTEGRIN BETA 1 (FIBRONECTIN RECEPTOR BETA)	5	2	-1.205	1.062E-01	2.745E-01
Q9WV80	SORTING NEXIN 1	13	4	-1.204	2.392E-02	9.969E-02
P16460	ARGININOSUCCINATE SYNTHETASE 1	99	9	-1.203	1.974E-04	2.311E-03
Q8CGK3	PROTEASE, SERINE, 15	19	7	-1.203	3.345E-01	5.570E-01
P23492	PURINE-NUCLEOSIDE PHOSPHORYLASE	10	4	-1.203	5.686E-01	7.581E-01

Q61207	PROSAPOSIN	6	1	-1.200	5.703E-03	3.574E-02
Q9D0K2	3-OXOACID COA TRANSFERASE 1	142	7	-1.199	1.432E-02	6.824E-02
Q9Z1D1	EUKARYOTIC TRANSLATION INITIATION FACTOR 3, SUBUNIT 4 (DELTA)	5	2	-1.197	3.730E-03	2.675E-02
Q9JKB3	COLD SHOCK DOMAIN PROTEIN A	8	3	-1.197	3.142E-01	5.332E-01
Q9EQH3	MATERNAL EMBRYONIC MESSAGE 3	7	1	-1.196	2.913E-01	5.198E-01
Q9CQM9	THIOREDOXIN-LIKE 2	5	2	-1.195	3.165E-01	5.348E-01
Q6PDM2	SPLICING FACTOR, ARGININE/SERINE-RICH 1 (ASF/SF2)	12	4	-1.194	1.540E-01	3.492E-01
Q9CPR4	RIBOSOMAL PROTEIN L17	7	1	-1.193	1.889E-02	8.446E-02
Q9DCT2	NADH DEHYDROGENASE (UBIQUINONE) FE-S PROTEIN 3	29	5	-1.192	3.987E-03	2.808E-02
Q8R164	BIPHENYL HYDROLASE-LIKE (SERINE HYDROLASE, BREAST EPITHELIAL MUCIN-ASSOCIATED ANTIGEN)	70	7	-1.191	6.850E-02	2.050E-01
P62821	RAB1, MEMBER RAS ONCOGENE FAMILY	7	1	-1.190	5.830E-02	1.849E-01
P51660	HYDROXYSTEROID (17-BETA) DEHYDROGENASE 4	21	6	-1.186	2.544E-01	4.764E-01
P36552	COPROPORPHYRINOGEN OXIDASE	6	3	-1.186	8.068E-02	2.294E-01
P28474	ALCOHOL DEHYDROGENASE 5 (CLASS III), CHI POLYPEPTIDE	9	1	-1.186	5.496E-02	1.786E-01
Q8VDJ3	DNA SEGMENT, CHR 1, ERATO DOI 101, EXPRESSED	9	5	-1.182	3.176E-01	5.356E-01
Q6IRU2	TROPOMYOSIN 4	13	4	-1.180	2.663E-01	4.931E-01
P62754	RIBOSOMAL PROTEIN S6	7	2	-1.180	8.493E-02	2.372E-01
Q922R8	PROTEIN DISULFIDE ISOMERASE ASSOCIATED 6	25	6	-1.179	2.655E-02	1.078E-01
Q9WUR2	PEROXISOMAL DELTA3, DELTA2-ENOYL-COENZYME A ISOMERASE	8	1	-1.178	4.296E-01	6.417E-01
Q9CXU9	EUKARYOTIC TRANSLATION INITIATION FACTOR 1B	13	2	-1.176	5.097E-01	7.069E-01
P58281	OPTIC ATROPHY 1 HOMOLOG (HUMAN)	18	4	-1.175	3.727E-01	5.945E-01
Q9DCV7	KERATIN COMPLEX 2, BASIC, GENE 7	9	2	-1.175	6.473E-01	8.127E-01
P97384	ANNEXIN A11	15	4	-1.173	2.495E-01	4.751E-01
P63101	TYROSINE 3-MONOOXYGENASE/TRYPTOPHAN 5-MONOOXYGENASE ACTIVATION PROTEIN, ZETA POLYPEPTIDE	45	5	-1.173	2.222E-02	9.508E-02
P31428	DIPEPTIDASE 1 (RENAL)	32	6	-1.172	4.293E-02	1.486E-01
P62717	RIBOSOMAL PROTEIN L18A	5	1	-1.170	1.139E-02	5.701E-02
Q9D1I5	METHYLMALONYL COA EPIMERASE	5	1	-1.170	4.727E-01	6.743E-01
P17156	HEAT SHOCK PROTEIN 2	17	2	-1.170	7.991E-03	4.576E-02
Q8QZT1	ACETYL-COENZYME A ACETYLTRANSFERASE 1	79	7	-1.169	1.007E-01	2.699E-01
Q9D0E1	HETEROGENEOUS NUCLEAR RIBONUCLEOPROTEIN M	13	6	-1.166	3.384E-01	5.588E-01
P35486	PYRUVATE DEHYDROGENASE E1 ALPHA 1	19	7	-1.163	3.993E-01	6.196E-01
Q9Z1Z0	VESICLE DOCKING PROTEIN	7	3	-1.161	2.076E-01	4.303E-01
P47738	ALDEHYDE DEHYDROGENASE 2, MITOCHONDRIAL	50	9	-1.159	4.820E-02	1.626E-01
P62835	RAS-RELATED PROTEIN-1A	34	1	-1.158	6.234E-03	3.847E-02
Q05421	CYTOCHROME P450, FAMILY 2, SUBFAMILY E, POLYPEPTIDE 1	8	2	-1.155	7.375E-02	2.166E-01
Q3ULD5	METHYLCROTONOYL-COENZYME A CARBOXYLASE 2 (BETA)	36	7	-1.155	3.659E-01	5.920E-01
Q8BVE3	EXPRESSED SEQUENCE AU022349	27	4	-1.152	2.127E-01	4.318E-01
Q9QYB1	CHLORIDE INTRACELLULAR CHANNEL 4 (MITOCHONDRIAL)	27	7	-1.151	1.042E-01	2.719E-01
Q920R6	ATPASE, H+ TRANSPORTING, LYSOSOMAL V0 SUBUNIT A4	8	3	-1.149	2.090E-01	4.299E-01

Q925B0	PRKC, APOPTOSIS, WT1, REGULATOR	7	3	-1.148	1.482E-01	3.419E-01
Q9D051	PYRUVATE DEHYDROGENASE (LIPOAMIDE) BETA	74	8	-1.146	1.593E-01	3.591E-01
Q9DCS3	MITOCHONDRIAL TRANS-2-ENOYL-COA REDUCTASE	9	1	-1.145	9.148E-02	2.511E-01
Q9Z2Y8	PROLINE SYNTHETASE CO-TRANSCRIBED	53	5	-1.144	2.923E-01	5.194E-01
P09242	ALKALINE PHOSPHATASE 2, LIVER	11	3	-1.144	1.791E-01	3.831E-01
P09103	PROLYL 4-HYDROXYLASE, BETA POLYPEPTIDE	46	9	-1.143	9.461E-03	5.089E-02
P20108	PEROXIREDOXIN 3	8	1	-1.143	3.569E-01	5.798E-01
Q61598	GUANOSINE DIPHOSPHATE (GDP) DISSOCIATION INHIBITOR 2	42	8	-1.141	1.013E-02	5.304E-02
Q91YI0	RIKEN CDNA 2510006M18 GENE	17	6	-1.140	4.166E-01	6.366E-01
P61759	VON HIPPEL-LINDAU BINDING PROTEIN 1	8	1	-1.140	1.862E-02	8.373E-02
Q6PDN3	MYOSIN, LIGHT POLYPEPTIDE KINASE	6	2	-1.139	1.816E-01	3.875E-01
P51174	ACETYL-COENZYME A DEHYDROGENASE, LONG-CHAIN	35	7	-1.139	4.943E-01	6.940E-01
P60867	RIBOSOMAL PROTEIN S20	9	1	-1.138	5.536E-01	7.494E-01
P62264	RIBOSOMAL PROTEIN S14	10	1	-1.136	8.387E-03	4.735E-02
Q8K4G5	ACTIN-BINDING LIM PROTEIN 1	7	2	-1.136	4.929E-01	6.932E-01
Q64010	V-CRK SARCOMA VIRUS CT10 ONCOGENE HOMOLOG (AVIAN)	8	2	-1.136	7.738E-01	8.953E-01
Q9CXW4	RIBOSOMAL PROTEIN L11	5	1	-1.135	1.172E-01	2.926E-01
P60843	EUKARYOTIC TRANSLATION INITIATION FACTOR 4A, PSEUDOGENE 4	5	1	-1.134	3.077E-01	5.268E-01
O55022	PROGESTERONE RECEPTOR MEMBRANE COMPONENT 1	10	2	-1.134	6.639E-01	8.244E-01
P55096	ATP-BINDING CASSETTE, SUB-FAMILY D (ALD), MEMBER 3	14	3	-1.133	4.869E-01	6.860E-01
Q8VC30	DIHYDROXYACETONE KINASE 2 HOMOLOG (YEAST)	51	9	-1.133	1.943E-01	4.092E-01
P53395	DIHYDROLIPOAMIDE BRANCHED CHAIN TRANSACYLASE E2	11	3	-1.132	4.119E-01	6.306E-01
Q9DBL7	COENZYME A SYNTHASE	7	2	-1.131	3.447E-02	1.294E-01
Q62261	SPECTRIN BETA 2	163	37	-1.131	2.412E-02	1.000E-01
P27659	RIBOSOMAL PROTEIN L3	14	4	-1.130	5.788E-01	7.679E-01
O35643	ADAPTOR PROTEIN COMPLEX AP-1, BETA 1 SUBUNIT	15	5	-1.130	1.296E-01	3.185E-01
P56389	CYTIDINE DEAMINASE	16	2	-1.129	4.716E-02	1.604E-01
P62960	Y BOX PROTEIN 1	7	1	-1.127	2.527E-01	4.756E-01
Q64727	VINCULIN	45	15	-1.127	2.980E-01	5.236E-01
P47911	RIBOSOMAL PROTEIN L6	6	3	-1.127	3.868E-01	6.097E-01
P21460	CYSTATIN C	7	1	-1.125	4.753E-03	3.206E-02
P60710	ACTIN, BETA, CYTOPLASMIC	159	5	-1.125	1.006E-01	2.706E-01
P20029	HEAT SHOCK 70KD PROTEIN 5 (GLUCOSE-REGULATED PROTEIN)	48	13	-1.123	3.125E-01	5.326E-01
Q99JY9	ARP3 ACTIN-RELATED PROTEIN 3 HOMOLOG (YEAST)	12	6	-1.121	3.462E-01	5.670E-01
P08113	TUMOR REJECTION ANTIGEN GP96	30	6	-1.120	1.313E-02	6.450E-02
Q02788	PROCOLLAGEN, TYPE VI, ALPHA 2	8	3	-1.120	1.741E-01	3.766E-01
Q9JLJ2	ALDEHYDE DEHYDROGENASE 9, SUBFAMILY A1	20	4	-1.119	3.478E-01	5.673E-01
Q3TC72	FUMARYLACETOACETATE HYDROLASE DOMAIN CONTAINING 2A	13	3	-1.119	4.311E-01	6.425E-01
Q9CYZ2	TUMOR PROTEIN D52-LIKE 2	6	2	-1.118	3.058E-01	5.269E-01
Q9CY58	SERPINE1 MRNA BINDING PROTEIN 1	5	1	-1.118	6.319E-03	3.869E-02
P21981	TRANSGLUTAMINASE 2, C POLYPEPTIDE	26	6	-1.116	1.337E-01	3.235E-01

Q9Z2I0	LEUCINE ZIPPER-EF-HAND CONTAINING TRANSMEMBRANE PROTEIN 1	24	10	-1.116	2.404E-01	4.655E-01
P29758	ORNITHINE AMINOTRANSFERASE	13	4	-1.115	6.935E-01	8.390E-01
Q9CQH7	RIKEN CDNA 4632412E09 GENE	7	2	-1.115	1.514E-01	3.463E-01
P97855	RAS-GTPASE-ACTIVATING PROTEIN SH3-DOMAIN BINDING PROTEIN	10	3	-1.114	2.717E-01	4.948E-01
Q3U0V1	KH-TYPE SPLICING REGULATORY PROTEIN	13	5	-1.114	4.703E-01	6.720E-01
Q9D964	GLYCINE AMIDINOTRANSFERASE (L-ARGININE:GLYCINE AMIDINOTRANSFERASE)	58	6	-1.113	2.701E-01	4.943E-01
O88844	ISOCITRATE DEHYDROGENASE 1 (NADP+), SOLUBLE	110	12	-1.111	3.763E-02	1.361E-01
Q99LP6	GRPE-LIKE 1, MITOCHONDRIAL	5	1	-1.110	6.059E-01	7.868E-01
Q6ZWV3	RIBOSOMAL PROTEIN 10	9	1	-1.108	4.179E-01	6.360E-01
P50396	GUANOSINE DIPHOSPHATE (GDP) DISSOCIATION INHIBITOR 1	12	4	-1.108	1.482E-01	3.410E-01
Q9DBG6	RIBOPHORIN II	7	2	-1.107	7.167E-01	8.541E-01
Q8VCI5	PEROXISOME BIOGENESIS FACTOR 19	5	2	-1.106	8.703E-01	9.400E-01
O08997	ATX1 (ANTIOXIDANT PROTEIN 1) HOMOLOG 1 (YEAST)	8	2	-1.105	8.026E-01	9.101E-01
P97429	ANNEXIN A4	20	5	-1.104	1.034E-01	2.726E-01
P48193	ERYTHROCYTE PROTEIN BAND 4.1	8	2	-1.103	4.345E-01	6.417E-01
P26039	TALIN 1	63	25	-1.101	1.655E-01	3.670E-01
P06151	LACTATE DEHYDROGENASE A	31	5	-1.100	4.232E-01	6.380E-01
Q8VCR7	ABHYDROLASE DOMAIN CONTAINING 14B	13	2	-1.100	2.399E-01	4.657E-01
Q99L45	EUKARYOTIC TRANSLATION INITIATION FACTOR 2, SUBUNIT 2 (BETA)	5	1	-1.100	4.094E-02	1.442E-01
Q91VM9	PYROPHOSPHATASE (INORGANIC) 2	24	5	-1.100	2.984E-01	5.209E-01
Q9CZ13	UBIQUINOL-CYTOCHROME C REDUCTASE CORE PROTEIN 1	101	12	-1.098	1.958E-01	4.102E-01
Q9QZD8	SOLUTE CARRIER FAMILY 25 (MITOCHONDRIAL CARRIER, DICARBOXYLATE TRANSPORTER), MEMBER 10	5	1	-1.098	7.263E-01	8.603E-01
P68254	TYROSINE 3-MONOOXYGENASE/TRYPTOPHAN 5-MONOOXYGENASE ACTIVATION PROTEIN, THETA POLYPEPTIDE	12	3	-1.095	7.808E-02	2.236E-01
Q7TPR4	ACTININ, ALPHA 1	28	12	-1.094	4.343E-01	6.437E-01
Q61081	CELL DIVISION CYCLE 37 HOMOLOG (S. CEREVISIAE)	12	2	-1.094	2.367E-01	4.640E-01
Q9EP89	LACTAMASE, BETA	6	1	-1.093	5.946E-01	7.797E-01
P16406	GLUTAMYL AMINOPEPTIDASE	17	6	-1.092	1.074E-01	2.766E-01
Q9DBP5	CYTIDYLATE KINASE	12	5	-1.090	6.014E-01	7.823E-01
P70670	NASCENT POLYPEPTIDE-ASSOCIATED COMPLEX ALPHA POLYPEPTIDE	6	1	-1.090	2.307E-01	4.534E-01
P40124	CAP, ADENYLATE CYCLASE-ASSOCIATED PROTEIN 1 (YEAST)	18	6	-1.090	1.417E-01	3.298E-01
Q9CR68	UBIQUINOL-CYTOCHROME C REDUCTASE, RIESKE IRON-SULFUR POLYPEPTIDE 1	12	1	-1.090	3.965E-01	6.201E-01
P19783	CYTOCHROME C OXIDASE SUBUNIT IV ISOFORM 1	38	4	-1.089	1.623E-01	3.619E-01
O35129	PROHIBITIN 2	69	4	-1.089	5.116E-02	1.683E-01
P14206	RIBOSOMAL PROTEIN SA	10	4	-1.089	3.969E-01	6.194E-01
Q8BH95	ENOYL COENZYME A HYDRATASE, SHORT CHAIN, 1, MITOCHONDRIAL	19	3	-1.089	5.674E-01	7.604E-01
Q9QYJ0	DNAJ (HSP40) HOMOLOG, SUBFAMILY A, MEMBER 2	7	3	-1.086	6.846E-01	8.295E-01
Q9CQF4	RIKEN CDNA 1700021F05 GENE	5	1	-1.086	8.076E-01	9.119E-01
Q9WV55	VESICLE-ASSOCIATED MEMBRANE PROTEIN, ASSOCIATED PROTEIN A	5	1	-1.086	5.743E-01	7.631E-01
Q9CPV4	RIKEN CDNA 2700085E05 GENE	21	5	-1.085	3.667E-01	5.897E-01

P08752	GUANINE NUCLEOTIDE BINDING PROTEIN, ALPHA INHIBITING 2	5	2	-1.085	6.800E-01	8.289E-01
P84244	H3 HISTONE, FAMILY 3A	13	1	-1.084	7.166E-01	8.552E-01
Q8BL66	EARLY ENDOSOME ANTIGEN 1	6	3	-1.083	2.872E-01	5.148E-01
Q9QUI0	RAS HOMOLOG GENE FAMILY, MEMBER A	10	1	-1.083	4.343E-01	6.426E-01
Q91X72	HEMOPEXIN	6	2	-1.082	8.973E-01	9.523E-01
Q922D8	METHYLENETETRAHYDROFOLATE DEHYDROGENASE (NADP+ DEPENDENT), METHENYLTETRAHYDROFOLATE CYCLOHYDROLASE, FORMYLTETRAHYDROFOLATE SYNTHASE	33	11	-1.079	3.905E-01	6.143E-01
Q7TMM9	TUBULIN, BETA 2A	104	10	-1.079	3.670E-02	1.334E-01
Q60875	RHO/RAC GUANINE NUCLEOTIDE EXCHANGE FACTOR (GEF) 2	6	2	-1.079	4.314E-01	6.418E-01
P16332	METHYLMALONYL-COENZYME A MUTASE	9	3	-1.078	5.495E-01	7.477E-01
Q9JKF1	IQ MOTIF CONTAINING GTPASE ACTIVATING PROTEIN 1	6	4	-1.078	8.583E-01	9.436E-01
O08756	3-HYDROXYACYL-COA DEHYDROGENASE TYPE II	15	3	-1.076	6.724E-01	8.247E-01
Q62422	OSTEOCLAST STIMULATING FACTOR 1	7	1	-1.076	2.485E-01	4.743E-01
Q9DCD0	RIKEN CDNA 0610042A05 GENE	16	2	-1.073	3.663E-01	5.902E-01
Q9ESG4	TRANSMEMBRANE PROTEIN 27	8	1	-1.073	3.928E-01	6.168E-01
P62315	SMALL NUCLEAR RIBONUCLEOPROTEIN D1	6	1	-1.072	8.193E-01	9.185E-01
Q8CHT0	ALDEHYDE DEHYDROGENASE 4 FAMILY, MEMBER A1	205	10	-1.072	8.543E-02	2.369E-01
Q9CR21	RIKEN CDNA 2310039H15 GENE	22	1	-1.071	6.668E-01	8.242E-01
P97351	RIBOSOMAL PROTEIN S3A	45	2	-1.071	1.053E-01	2.731E-01
P97371	PROTEASOME (PROSOME, MACROPAIN) 28 SUBUNIT, ALPHA	13	3	-1.067	7.406E-01	8.708E-01
Q61937	NUCLEOPHOSMIN 1	11	2	-1.066	4.636E-01	6.697E-01
Q61768	KINESIN FAMILY MEMBER 5B	11	4	-1.065	6.458E-01	8.134E-01
Q99MZ7	RIKEN CDNA 2400003B18 GENE	14	2	-1.065	7.147E-01	8.543E-01
P08226	APOLIPOPROTEIN E	5	1	-1.065	6.252E-01	8.027E-01
Q8CGB3	UVEAL AUTOANTIGEN WITH COILED-COIL DOMAINS AND ANKYRIN REPEATS	10	1	-1.063	3.978E-01	6.196E-01
Q9JII6	ALDO-KETO REDUCTASE FAMILY 1, MEMBER A4 (ALDEHYDE REDUCTASE)	139	4	-1.061	2.033E-01	4.248E-01
Q9QZD9	EUKARYOTIC TRANSLATION INITIATION FACTOR 3, SUBUNIT 2 (BETA)	5	1	-1.059	4.621E-01	6.699E-01
Q9Z0J0	NIEMANN PICK TYPE C2	5	1	-1.058	1.364E-01	3.289E-01
P19253	RIBOSOMAL PROTEIN L13A	5	1	-1.058	7.250E-01	8.600E-01
P05213	TUBULIN, ALPHA 2	7	1	-1.057	5.560E-01	7.514E-01
Q9QYC0	ADDUCIN 1 (ALPHA)	12	6	-1.057	6.582E-01	8.212E-01
Q9CQV8	TYROSINE 3-MONOOXYGENASE/TRYPTOPHAN 5-MONOOXYGENASE ACTIVATION PROTEIN, BETA POLYPEPTIDE	22	2	-1.057	4.598E-01	6.692E-01
Q91VD9	NADH DEHYDROGENASE (UBIQUINONE) FE-S PROTEIN 1	60	11	-1.057	5.874E-01	7.741E-01
P62962	PROFILIN 1	16	2	-1.056	4.005E-01	6.203E-01
Q8VCA8	SECERNIN 2	11	2	-1.056	7.435E-01	8.716E-01
P62301	RIBOSOMAL PROTEIN S13	6	1	-1.056	2.511E-01	4.770E-01
Q9JHW4	EUKARYOTIC ELONGATION FACTOR, SELENOCYSTEINE-TRNA-SPECIFIC	6	2	-1.055	5.863E-01	7.740E-01
P70404	ISOCITRATE DEHYDROGENASE 3 (NAD+), GAMMA	13	1	-1.055	8.361E-01	9.282E-01
Q9D0M3	CYTOCHROME C-1	92	4	-1.053	2.479E-01	4.756E-01

Q9D8E6	RIBOSOMAL PROTEIN L4	8	3	-1.053	8.477E-01	9.372E-01
Q01405	SEC23A (S. CEREVISIAE)	5	1	-1.052	6.743E-01	8.258E-01
P29699	ALPHA-2-HS-GLYCOPROTEIN	15	3	-1.052	8.620E-01	9.438E-01
Q9D0F9	PHOSPHOGLUCOMUTASE 2	15	4	-1.051	5.881E-01	7.737E-01
O70250	PHOSPHOGLYCERATE MUTASE 2	6	2	-1.051	4.495E-01	6.578E-01
Q62448	EUKARYOTIC TRANSLATION INITIATION FACTOR 4, GAMMA 2	5	1	-1.050	6.433E-01	8.127E-01
P61982	3-MONOOXYGENASE/TRYPTOPHAN 5-MONOOXYGENASE ACTIVATION PROTEIN, GAMMA POLYPEPTIDE	8	2	-1.049	5.570E-01	7.514E-01
Q9CY64	RIKEN CDNA 0610006A11 GENE	5	2	-1.049	6.556E-01	8.205E-01
P05064	ALDOLASE 1, A ISOFORM	35	5	-1.048	4.958E-01	6.948E-01
P14869	ACIDIC RIBOSOMAL PHOSPHOPROTEIN P0	7	3	-1.048	7.121E-01	8.524E-01
Q7TNV0	RIKEN CDNA 1810019E15 GENE	5	1	-1.047	8.248E-01	9.221E-01
Q8BMF4	DIHYDROLIPOAMIDE S-ACETYLTRANSFERASE (E2 COMPONENT OF PYRUVATE DEHYDROGENASE COMPLEX)	38	6	-1.047	1.038E-01	2.718E-01
Q7TMK9	NS1-ASSOCIATED PROTEIN 1-LIKE	7	2	-1.045	6.256E-01	8.018E-01
O08709	PEROXIREDOXIN 6	31	6	-1.044	6.547E-01	8.207E-01
Q8BH86	RIKEN CDNA 9030617O03 GENE	26	6	-1.044	6.144E-01	7.939E-01
Q923I7	LOW AFFINITY SODIUM-DEPENDENT GLUCOSE COTRANSPORTER	12	3	-1.044	8.678E-01	9.424E-01
Q9CQN1	TNF RECEPTOR-ASSOCIATED PROTEIN 1	11	4	-1.044	6.645E-01	8.239E-01
P06745	GLUCOSE PHOSPHATE ISOMERASE 1	9	1	-1.043	6.652E-01	8.235E-01
P62492	RAB11A, MEMBER RAS ONCOGENE FAMILY	6	2	-1.040	8.511E-01	9.396E-01
Q8BTM8	FILAMIN, ALPHA	35	15	-1.040	7.682E-01	8.940E-01
Q9CWS0	DIMETHYLARGININE DIMETHYLAMINOHYDROLASE 1	29	5	-1.040	4.677E-01	6.720E-01
Q9D2V7	CORONIN 7	5	2	-1.039	8.328E-01	9.272E-01
P05202	GLUTAMATE OXALOACETATE TRANSAMINASE 2, MITOCHONDRIAL	75	4	-1.039	3.384E-01	5.578E-01
P14824	ANNEXIN A6	12	6	-1.039	7.692E-01	8.939E-01
P80316	CHAPERONIN SUBUNIT 5 (EPSILON)	6	2	-1.039	8.685E-01	9.406E-01
Q9R0P5	DESTRIN	30	3	-1.036	4.692E-01	6.730E-01
P62897	CYTOCHROME C, SOMATIC	46	4	-1.036	5.213E-01	7.179E-01
P28271	ACONITASE 1	50	11	-1.034	6.353E-01	8.052E-01
Q9DB15	MITOCHONDRIAL RIBOSOMAL PROTEIN L12	6	2	-1.034	6.562E-01	8.200E-01
P48758	CARBONYL REDUCTASE 1	14	3	-1.034	7.904E-01	9.014E-01
Q9D1Q6	THIOREDOXIN DOMAIN CONTAINING 4 (ENDOPLASMIC RETICULUM)	5	2	-1.033	8.827E-01	9.483E-01
Q922Q1	MOCO SULPHURASE C-TERMINAL DOMAIN CONTAINING 2	30	4	-1.033	8.248E-01	9.234E-01
Q9QXX4	SOLUTE CARRIER FAMILY 25 (MITOCHONDRIAL CARRIER, ADENINE NUCLEOTIDE TRANSLOCATOR), MEMBER 13	14	5	-1.033	6.013E-01	7.833E-01
P63017	HEAT SHOCK PROTEIN 8	51	9	-1.032	6.457E-01	8.146E-01
P18760	COFILIN 1, NON-MUSCLE	13	2	-1.031	5.015E-01	7.004E-01
Q9R112	SULFIDE QUINONE REDUCTASE-LIKE (YEAST)	8	2	-1.031	8.814E-01	9.494E-01
O35326	SPLICING FACTOR, ARGININE/SERINE-RICH 5 (SRP40, HRS)	9	1	-1.031	8.518E-01	9.391E-01
Q6WVG3	EXPRESSED SEQUENCE AW538430	5	3	-1.031	8.908E-01	9.518E-01

Q9DBT9	DIMETHYLGLYCINE DEHYDROGENASE PRECURSOR	9	3	-1.029	9.554E-01	9.762E-01
P97742	CARNITINE PALMITOYLTRANSFERASE 1A, LIVER	11	3	-1.028	8.536E-01	9.398E-01
O09173	HOMOGENTISATE 1, 2-DIOXYGENASE	19	4	-1.027	6.761E-01	8.266E-01
Q9WVM8	AMINOADIPATE AMINOTRANSFERASE	5	1	-1.027	7.712E-01	8.949E-01
P50247	S-ADENOSYLHOMOCYSTEINE HYDROLASE	29	6	-1.027	7.983E-01	9.065E-01
P97807	FUMARATE HYDRATASE 1	24	7	-1.026	7.279E-01	8.596E-01
P70290	MEMBRANE PROTEIN, PALMITOYLATED	9	4	-1.026	9.152E-01	9.624E-01
Q8K2B3	SUCCINATE DEHYDROGENASE COMPLEX, SUBUNIT A, FLAVOPROTEIN (FP)	144	13	-1.025	6.634E-01	8.251E-01
Q9CZ30	RIKEN CDNA 2810409H07 GENE	11	3	-1.025	7.780E-01	8.975E-01
Q6P1B1	X-PROLYL AMINOPEPTIDASE (AMINOPEPTIDASE P) 1, SOLUBLE	9	4	-1.025	7.336E-01	8.638E-01
Q9WTP7	ADENYLATE KINASE 3	16	5	-1.024	8.433E-01	9.350E-01
P20152	VIMENTIN	20	6	-1.024	8.470E-01	9.377E-01
P55264	ADENOSINE KINASE	12	1	-1.024	9.184E-01	9.619E-01
Q99MN9	PROPIONYL COENZYME A CARBOXYLASE, BETA POLYPEPTIDE	33	10	-1.022	8.161E-01	9.162E-01
Q9JLB4	CUBILIN (INTRINSIC FACTOR-COBALAMIN RECEPTOR)	34	16	-1.022	8.617E-01	9.447E-01
P09411	PHOSPHOGLYCERATE KINASE 1	243	13	-1.022	6.297E-01	8.019E-01
P62843	RIBOSOMAL PROTEIN S15	16	1	-1.021	8.658E-01	9.428E-01
Q99L13	3-HYDROXYISOBUTYRATE DEHYDROGENASE	44	7	-1.020	7.215E-01	8.572E-01
P17563	SELENIUM BINDING PROTEIN 1	92	9	-1.020	6.813E-01	8.279E-01
Q62093	SPLICING FACTOR, ARGININE/SERINE-RICH 10	12	1	-1.019	7.330E-01	8.644E-01
Q9CQM5	THIOREDOXIN-LIKE 5	8	1	-1.019	8.690E-01	9.399E-01
Q64331	MYOSIN VI	21	7	-1.018	7.974E-01	9.068E-01
P15864	HISTONE 1, H1C	9	1	-1.018	8.841E-01	9.484E-01
Q9CQ92	FISSION 1 (MITOCHONDRIAL OUTER MEMBRANE) HOMOLOG (YEAST)	5	1	-1.018	8.926E-01	9.512E-01
P97449	ALANYL (MEMBRANE) AMINOPEPTIDASE	28	8	-1.017	8.961E-01	9.524E-01
Q99JB2	STOMATIN (EPB7.2)-LIKE 2	9	1	-1.017	8.819E-01	9.487E-01
Q9WVA4	TRANSGELIN 2	64	6	-1.016	8.596E-01	9.438E-01
Q9D6J6	NADH DEHYDROGENASE (UBIQUINONE) FLAVOPROTEIN 2	38	5	-1.016	8.308E-01	9.263E-01
Q6PGL7	DNA SEGMENT, CHR 6, WAYNE STATE UNIVERSITY 116, EXPRESSED	5	4	-1.015	9.655E-01	9.853E-01
O55143	ATPASE, CA++ TRANSPORTING, CARDIAC MUSCLE, SLOW TWITCH 2	9	3	-1.015	9.062E-01	9.567E-01
Q07417	ACYL-COENZYME A DEHYDROGENASE, SHORT CHAIN	25	6	-1.015	9.173E-01	9.620E-01
Q99KJ8	DYNACTIN 2	10	5	-1.013	9.286E-01	9.675E-01
Q9JLZ3	AU RNA BINDING PROTEIN/ENOYL-COENZYME A HYDRATASE	5	3	-1.013	9.447E-01	9.766E-01
Q99KR3	LACTAMASE, BETA 2	14	3	-1.011	8.925E-01	9.523E-01
P62073	TRANSLOCASE OF INNER MITOCHONDRIAL MEMBRANE 10 HOMOLOG (YEAST)	6	1	-1.011	9.070E-01	9.562E-01
P56135	ATP SYNTHASE, H+ TRANSPORTING, MITOCHONDRIAL F0 COMPLEX, SUBUNIT F, ISOFORM 2	8	1	-1.011	9.377E-01	9.719E-01
Q8BWF0	ALDHEHYDE DEHYDROGENASE FAMILY 5, SUBFAMILY A1	19	6	-1.010	9.453E-01	9.760E-01
Q8VCN5	CYSTATHIONASE (CYSTATHIONINE GAMMA-LYASE)	12	2	-1.010	8.640E-01	9.447E-01
Q80X90	EXPRESSED SEQUENCE AL024016	84	27	-1.008	8.650E-01	9.445E-01
Q8BFW7	LIM DOMAIN CONTAINING PREFERRED TRANSLOCATION PARTNER IN LIPOMA	10	4	-1.008	9.338E-01	9.703E-01

Q62468	VILLIN 1	75	14	-1.008	9.089E-01	9.570E-01
P62242	RIBOSOMAL PROTEIN S8	15	4	-1.008	9.159E-01	9.619E-01
P14733	LAMIN B1	13	3	-1.007	9.350E-01	9.704E-01
Q3UGR5	RIKEN CDNA 0610039H12 GENE	7	1	-1.006	7.861E-01	8.991E-01
P67984	RIBOSOMAL PROTEIN L22	9	1	-1.005	9.807E-01	9.944E-01
Q61739	INTEGRIN ALPHA 6	7	1	-1.005	9.456E-01	9.737E-01
P99029	PEROXISOMAL MEMBRANE PROTEIN 20	164	7	-1.004	9.192E-01	9.615E-01
Q9JKR6	HYPOXIA UP-REGULATED 1	13	6	-1.003	9.703E-01	9.889E-01
P19157	GLUTATHIONE S-TRANSFERASE, PI 1	14	3	-1.003	9.546E-01	9.767E-01
Q78IK4	RIKEN CDNA 9430083G14 GENE	9	5	-1.003	9.855E-01	9.980E-01
Q9Z0X1	PROGRAMMED CELL DEATH 8	46	11	-1.003	9.540E-01	9.773E-01
O35658	COMPLEMENT COMPONENT 1, Q SUBCOMPONENT BINDING PROTEIN	6	1	-1.002	9.881E-01	9.969E-01
P26645	MYRISTOYLATED ALANINE RICH PROTEIN KINASE C SUBSTRATE	5	2	-1.001	9.976E-01	1.000E+00
Q921I1	TRANSFERRIN	21	7	-1.001	9.901E-01	9.963E-01
Q9CQH3	NADH DEHYDROGENASE (UBIQUINONE) 1 BETA SUBCOMPLEX, 5	7	1	-1.001	9.952E-01	9.990E-01
P47962	RIBOSOMAL PROTEIN L5	17	4	-1.000	9.987E-01	9.987E-01
P62737	ACTIN, ALPHA 2, SMOOTH MUSCLE, AORTA	150	8	1.000	9.978E-01	9.990E-01
P58252	EUKARYOTIC TRANSLATION ELONGATION FACTOR 2	30	10	1.001	9.872E-01	9.972E-01
P80314	CHAPERONIN SUBUNIT 2 (BETA)	14	4	1.002	9.920E-01	9.971E-01
Q8VI36	PAXILLIN	6	1	1.002	9.887E-01	9.962E-01
P12970	RIBOSOMAL PROTEIN L7A	8	2	1.002	9.864E-01	9.977E-01
Q9Z0P4	PARALEMMIN	14	4	1.003	9.724E-01	9.885E-01
P63038	HEAT SHOCK PROTEIN 1 (CHAPERONIN)	510	17	1.005	9.026E-01	9.541E-01
Q8CIN4	P21 (CDKN1A)-ACTIVATED KINASE 2	5	1	1.005	8.935E-01	9.508E-01
Q91ZA3	EXPRESSED SEQUENCE C79630	41	10	1.005	9.518E-01	9.775E-01
P62259	TYROSINE 3-MONOOXYGENASE/TRYPTOPHAN 5-MONOOXYGENASE ACTIVATION PROTEIN, EPSILON POLYPEPTIDE	23	6	1.005	9.021E-01	9.549E-01
P26231	CATENIN (CADHERIN ASSOCIATED PROTEIN), ALPHA 1	31	6	1.005	9.534E-01	9.780E-01
Q7TMS5	ATP-BINDING CASSETTE, SUB-FAMILY G (WHITE), MEMBER 2	6	1	1.005	9.718E-01	9.892E-01
Q80XL6	ACYL-COENZYME A DEHYDROGENASE FAMILY, MEMBER 11	5	2	1.006	9.802E-01	9.952E-01
O88428	3'-PHOSPHOADENOSINE 5'-PHOSPHOSULFATE SYNTHASE 2	77	8	1.006	9.416E-01	9.746E-01
Q64442	SORBITOL DEHYDROGENASE	90	7	1.009	8.766E-01	9.455E-01
Q8VCH0	ACETYL-COENZYME A ACYLTRANSFERASE 1B	60	2	1.010	8.894E-01	9.516E-01
Q9WTX5	S-PHASE KINASE-ASSOCIATED PROTEIN 1A	7	1	1.010	9.263E-01	9.664E-01
Q9Z2I9	SUCCINATE-COENZYME A LIGASE, ADP-FORMING, BETA SUBUNIT	62	6	1.011	8.658E-01	9.440E-01
P15532	EXPRESSED IN NON-METASTATIC CELLS 1, PROTEIN	48	5	1.012	7.800E-01	8.972E-01
P16546	SPECTRIN ALPHA 2	207	51	1.013	7.805E-01	8.965E-01
Q64516	GLYCEROL KINASE	23	4	1.014	9.454E-01	9.748E-01
P16125	LACTATE DEHYDROGENASE B	90	6	1.014	8.056E-01	9.122E-01
Q6ZQ38	CULLIN ASSOCIATED AND NEDDYLATION DISASSOCIATED 1	6	2	1.015	9.220E-01	9.632E-01
Q8CG76	ALDO-KETO REDUCTASE FAMILY 7, MEMBER A5 (AFLATOXIN ALDEHYDE REDUCTASE)	9	3	1.017	9.495E-01	9.765E-01

Q9QXZ0	MICROTUBULE-ACTIN CROSSLINKING FACTOR 1	6	4	1.018	8.862E-01	9.494E-01
Q9CQ62	2,4-DIENOYL COA REDUCTASE 1, MITOCHONDRIAL	75	5	1.019	7.961E-01	9.066E-01
Q3UPL0	SEC31-LIKE 1 (S. CEREVISIAE)	5	2	1.019	8.150E-01	9.176E-01
Q60932	VOLTAGE-DEPENDENT ANION CHANNEL 1	412	9	1.021	5.860E-01	7.749E-01
Q9DCJ5	NADH DEHYDROGENASE (UBIQUINONE) 1 ALPHA SUBCOMPLEX, 8	11	3	1.021	6.990E-01	8.417E-01
P31786	DIAZEPAM BINDING INHIBITOR	12	1	1.021	8.677E-01	9.436E-01
Q8BG05	RIKEN CDNA 2610510D13 GENE	66	3	1.021	5.002E-01	6.997E-01
Q68FD5	CLATHRIN, HEAVY POLYPEPTIDE (HC)	85	21	1.022	7.456E-01	8.728E-01
P62702	RIBOSOMAL PROTEIN S4, X-LINKED	14	3	1.022	7.620E-01	8.881E-01
P34884	MACROPHAGE MIGRATION INHIBITORY FACTOR	11	1	1.022	9.293E-01	9.670E-01
P38647	HEAT SHOCK PROTEIN 9A	48	10	1.023	6.423E-01	8.129E-01
O55029	COATOMER PROTEIN COMPLEX, SUBUNIT BETA 2 (BETA PRIME)	5	1	1.023	6.979E-01	8.417E-01
P14152	MALATE DEHYDROGENASE 1, NAD (SOLUBLE)	58	9	1.024	7.815E-01	8.964E-01
Q9DCS9	NADH DEHYDROGENASE (UBIQUINONE) 1 BETA SUBCOMPLEX, 10	15	2	1.025	7.272E-01	8.602E-01
Q8CAQ8	INNER MEMBRANE PROTEIN, MITOCHONDRIAL	33	11	1.027	6.718E-01	8.252E-01
Q9CQC9	SAR1 GENE HOMOLOG B (S. CEREVISIAE)	5	1	1.027	7.020E-01	8.440E-01
Q9CR51	ATPASE, H+ TRANSPORTING, LYSOSOMAL V1 SUBUNIT G1	20	2	1.028	7.716E-01	8.940E-01
Q9CR61	NADH DEHYDROGENASE (UBIQUINONE) 1 BETA SUBCOMPLEX, 7	23	2	1.030	5.702E-01	7.590E-01
Q60597	OXOGLUTARATE DEHYDROGENASE (LIPOAMIDE)	109	16	1.031	4.200E-01	6.355E-01
O89017	LEGUMAIN	17	2	1.032	8.273E-01	9.237E-01
P62334	PROTEASOME (PROSOME, MACROPAIN) 26S SUBUNIT, ATPASE, 6	9	4	1.034	8.984E-01	9.522E-01
Q99KQ4	PRE-B-CELL COLONY-ENHANCING FACTOR 1	14	3	1.035	7.413E-01	8.703E-01
P00920	CARBONIC ANHYDRASE 2	38	5	1.036	7.181E-01	8.545E-01
Q8R1F5	HYDROXYPYRUVATE ISOMERASE HOMOLOG (E. COLI)	7	1	1.036	8.083E-01	9.113E-01
Q99020	HETEROGENEOUS NUCLEAR RIBONUCLEOPROTEIN A/B	8	1	1.037	7.747E-01	8.950E-01
Q61879	MYOSIN HEAVY CHAIN 10, NON-MUSCLE	52	18	1.037	7.073E-01	8.492E-01
P35282	RAB21, MEMBER RAS ONCOGENE FAMILY	5	1	1.039	8.345E-01	9.277E-01
Q9D358	ACID PHOSPHATASE 1, SOLUBLE	5	1	1.039	6.676E-01	8.239E-01
Q8VE37	REGULATOR OF CHROMOSOME CONDENSATION 1	7	3	1.039	8.058E-01	9.111E-01
Q61425	L-3-HYDROXYACYL-COENZYME A DEHYDROGENASE, SHORT CHAIN	66	3	1.040	6.690E-01	8.230E-01
Q9DC69	NADH DEHYDROGENASE (UBIQUINONE) 1 ALPHA SUBCOMPLEX, 9	44	2	1.041	5.970E-01	7.790E-01
Q99PL5	RIKEN CDNA 5730465C04 GENE	14	6	1.041	7.788E-01	8.972E-01
Q9WVT6	CARBONIC ANHYDRASE 14	5	1	1.042	7.826E-01	8.964E-01
O70251	EUKARYOTIC TRANSLATION ELONGATION FACTOR 1 BETA 2	17	2	1.043	6.803E-01	8.280E-01
Q9JHU4	DYNEIN CYTOPLASMIC 1 HEAVY CHAIN 1	18	11	1.043	4.849E-01	6.844E-01
O08749	DIHYDROLIPOAMIDE DEHYDROGENASE	103	8	1.045	5.125E-01	7.082E-01
P01027	COMPLEMENT COMPONENT 3	12	4	1.045	4.089E-01	6.296E-01
Q64475	HISTONE 1, H2BB	79	1	1.046	3.383E-01	5.599E-01
Q9Z0S1	BISPHOSPHATE 3'-NUCLEOTIDASE 1	24	5	1.048	6.124E-01	7.939E-01
Q91WN4	KYNURENINE 3-MONOOXYGENASE (KYNURENINE 3-HYDROXYLASE)	7	2	1.048	5.146E-01	7.099E-01
Q9D172	DNA SEGMENT, CHR 10, JOHNS HOPKINS UNIVERSITY 81 EXPRESSED	10	3	1.049	7.875E-01	8.994E-01

Q99LY9	EXPRESSED SEQUENCE AI256693	12	1	1.049	5.931E-01	7.791E-01
O88342	WD REPEAT DOMAIN 1	5	1	1.051	7.532E-01	8.791E-01
P56399	UBIQUITIN SPECIFIC PEPTIDASE 5 (ISOPEPTIDASE T)	9	4	1.054	7.105E-01	8.518E-01
Q91ZJ5	UDP-GLUCOSE PYROPHOSPHORYLASE 2	5	3	1.054	8.681E-01	9.415E-01
P30275	CREATINE KINASE, MITOCHONDRIAL 1, UBIQUITOUS	18	3	1.054	6.588E-01	8.206E-01
Q9D898	ACTIN RELATED PROTEIN 2/3 COMPLEX, SUBUNIT 5-LIKE	6	1	1.054	7.493E-01	8.758E-01
Q9WUM5	SUCCINATE-COA LIGASE, GDP-FORMING, ALPHA SUBUNIT	28	2	1.056	3.356E-01	5.577E-01
O09044	SYNAPTOSOMAL-ASSOCIATED PROTEIN 23	9	2	1.056	4.360E-01	6.427E-01
O70133	ATP-DEPENDENT RNA HELICASE A	11	1	1.058	4.172E-01	6.361E-01
Q9JIL4	PDZ DOMAIN CONTAINING 1	50	10	1.061	4.551E-01	6.634E-01
P29341	POLY A BINDING PROTEIN, CYTOPLASMIC 1	5	2	1.061	8.156E-01	9.170E-01
Q8R4N0	CITRATE LYASE BETA LIKE	10	3	1.061	6.159E-01	7.946E-01
P62075	TRANSLOCASE OF INNER MITOCHONDRIAL MEMBRANE 13 HOMOLOG (YEAST)	6	1	1.062	6.288E-01	8.022E-01
P09405	NUCLEOLIN	14	4	1.062	5.338E-01	7.301E-01
P26040	VILLIN 2	145	9	1.062	1.300E-01	3.175E-01
Q99JW2	RIKEN CDNA 1110014J22 GENE	9	3	1.063	5.956E-01	7.798E-01
Q9QXD6	FRUCTOSE BISPHOSPHATASE 1	163	10	1.065	2.669E-01	4.918E-01
Q99PT1	EXPRESSED SEQUENCE C87222	18	3	1.066	3.860E-01	6.108E-01
P00493	HYPOXANTHINE GUANINE PHOSPHORIBOSYL TRANSFERASE 1	5	2	1.067	4.407E-01	6.485E-01
Q05793	PERLECAN (HEPARAN SULFATE PROTEOGLYCAN 2)	37	13	1.068	2.740E-01	4.979E-01
Q91VR2	ATP SYNTHASE, H+ TRANSPORTING, MITOCHONDRIAL F1 COMPLEX, GAMMA POLYPEPTIDE 1	60	3	1.068	2.807E-01	5.055E-01
Q6A4J8	RIKEN CDNA 2210010O09 GENE	6	1	1.069	6.797E-01	8.298E-01
Q8VIJ6	SPLICING FACTOR PROLINE/GLUTAMINE RICH (POLYPYRIMIDINE TRACT BINDING PROTEIN ASSOCIATED)	10	2	1.070	5.662E-01	7.600E-01
P07356	ANNEXIN A2	10	3	1.072	1.075E-01	2.762E-01
P62991	RIBOSOMAL PROTEIN S27A	12	1	1.072	3.031E-01	5.233E-01
P68369	TUBULIN, ALPHA 1	56	7	1.072	1.594E-01	3.585E-01
Q9DC70	NADH DEHYDROGENASE (UBIQUINONE) FE-S PROTEIN 7	9	2	1.073	6.245E-01	8.030E-01
P17225	POLYPYRIMIDINE TRACT BINDING PROTEIN 1	11	3	1.073	5.676E-01	7.594E-01
Q91YR1	PROTEIN TYROSINE KINASE 9	7	3	1.074	2.288E-01	4.541E-01
P50518	VATPASE, H+ TRANSPORTING, LYSOSOMAL V1 SUBUNIT E1	35	4	1.078	2.517E-01	4.758E-01
P26043	RADIXIN	14	1	1.078	2.702E-01	4.934E-01
P62137	PROTEIN PHOSPHATASE 1, CATALYTIC SUBUNIT, ALPHA ISOFORM	10	3	1.078	4.830E-01	6.854E-01
Q8BH00	ALDEHYDE DEHYDROGENASE 8 FAMILY, MEMBER A1	37	8	1.079	2.744E-01	4.976E-01
Q9DCU9	RIKEN CDNA 0610010D20 GENE	9	1	1.080	2.887E-01	5.165E-01
Q9WV92	ERYTHROCYTE PROTEIN BAND 4.1-LIKE 3	24	6	1.082	4.472E-01	6.556E-01
P62908	RIBOSOMAL PROTEIN S3	16	5	1.083	3.399E-01	5.590E-01
Q9QZQ1	MYELOID/LYMPHOID OR MIXED LINEAGE-LEUKEMIA TRANSLOCATION TO 4 HOMOLOG (DROSOPHILA)	6	3	1.083	5.996E-02	1.887E-01
Q921G7	ELECTRON TRANSFERRING FLAVOPROTEIN, DEHYDROGENASE	18	3	1.084	4.197E-01	6.363E-01

Q6P9R2	RIKEN CDNA 2810422B09 GENE	5	1	1.085	5.529E-01	7.498E-01
P50516	ATPASE, H+ TRANSPORTING, LYSOSOMAL V1 SUBUNIT A	120	12	1.085	9.997E-02	2.697E-01
Q99JR1	SIDEROFLEXIN 1	13	3	1.086	3.149E-01	5.334E-01
Q9D8B4	NADH DEHYDROGENASE (UBIQUINONE) 1 ALPHA SUBCOMPLEX 11	6	1	1.089	5.264E-01	7.237E-01
Q9Z1W8	ATPASE, H+/K+ TRANSPORTING, NONGASTRIC, ALPHA POLYPEPTIDE	14	2	1.089	2.795E-01	5.045E-01
Q8BH59	SOLUTE CARRIER FAMILY 25 (MITOCHONDRIAL CARRIER, ARALAR), MEMBER 12	15	6	1.089	4.194E-01	6.371E-01
Q9JHJ0	TROPOMODULIN 3	5	1	1.089	6.243E-01	8.041E-01
Q61941	NICOTINAMIDE NUCLEOTIDE TRANSHYDROGENASE	22	9	1.093	2.991E-01	5.211E-01
P06728	APOLIPOPROTEIN A-IV	6	1	1.093	4.832E-01	6.844E-01
P47199	CRYSTALLIN, ZETA	44	8	1.094	2.222E-01	4.466E-01
Q91V76	RIKEN CDNA 4931406C07 GENE	73	5	1.095	2.550E-01	4.765E-01
Q9DBG3	ADAPTOR-RELATED PROTEIN COMPLEX 2, BETA 1 SUBUNIT	6	1	1.096	2.586E-01	4.809E-01
Q8BGQ7	ALANYL-TRNA SYNTHETASE	7	3	1.096	6.825E-01	8.281E-01
O88322	NIDOGEN 2	11	4	1.099	6.266E-01	8.006E-01
P26041	MOESIN	14	2	1.101	9.847E-02	2.666E-01
Q03265	ATP SYNTHASE, H+ TRANSPORTING, MITOCHONDRIAL F1 COMPLEX, ALPHA SUBUNIT, ISOFORM 1	745	13	1.102	2.518E-10	1.002E-08
P27546	MICROTUBULE-ASSOCIATED PROTEIN 4	23	4	1.102	5.115E-02	1.690E-01
Q8R086	SULFITE OXIDASE	19	3	1.103	4.804E-01	6.841E-01
P62806	HISTONE 1, H4H	29	4	1.104	4.064E-01	6.270E-01
P21278	GUANINE NUCLEOTIDE BINDING PROTEIN, ALPHA 11	6	1	1.104	1.874E-01	3.978E-01
Q8R1I1	RIKEN CDNA 1110020P15 GENE	11	1	1.105	1.647E-01	3.663E-01
Q8VDD5	MYOSIN, HEAVY POLYPEPTIDE 9, NON-MUSCLE	139	31	1.107	4.271E-02	1.485E-01
Q9Z2I8	SUCCINATE-COENZYME A LIGASE, GDP-FORMING, BETA SUBUNIT	62	6	1.107	1.083E-01	2.772E-01
Q78PY7	EXPRESSED SEQUENCE AL033314	10	4	1.110	6.463E-01	8.127E-01
Q99LC5	ELECTRON TRANSFERRING FLAVOPROTEIN, ALPHA POLYPEPTIDE	423	10	1.112	2.304E-05	3.986E-04
P08249	MALATE DEHYDROGENASE 2, NAD (MITOCHONDRIAL)	143	12	1.113	2.066E-02	9.038E-02
Q8VEM8	SOLUTE CARRIER FAMILY 25 (MITOCHONDRIAL CARRIER, PHOSPHATE CARRIER), MEMBER 3	12	3	1.114	3.661E-01	5.912E-01
Q80W22	CDNA SEQUENCE BC051244	10	1	1.115	1.388E-01	3.298E-01
O08810	ELONGATION FACTOR TU GTP BINDING DOMAIN CONTAINING 2	5	2	1.117	6.132E-01	7.936E-01
Q64433	HEAT SHOCK PROTEIN 1 (CHAPERONIN 10)	21	1	1.117	3.867E-01	6.107E-01
P54071	ISOCITRATE DEHYDROGENASE 2 (NADP+), MITOCHONDRIAL	158	8	1.119	1.036E-01	2.721E-01
P10107	ANNEXIN A1	7	2	1.119	3.825E-01	6.077E-01
Q8BGH2	SORTING AND ASSEMBLY MACHINERY COMPONENT 50 HOMOLOG (S. CEREVISIAE)	9	2	1.121	5.581E-01	7.517E-01
P61979	HETEROGENEOUS NUCLEAR RIBONUCLEOPROTEIN K	44	9	1.121	2.556E-01	4.766E-01
Q05920	PYRUVATE CARBOXYLASE	246	16	1.122	2.724E-03	2.126E-02
O70492	SORTING NEXIN 3	8	2	1.122	2.983E-01	5.219E-01
P47791	GLUTATHIONE REDUCTASE 1	7	4	1.123	2.978E-01	5.245E-01
P05784	KERATIN COMPLEX 1, ACIDIC, GENE 18	11	4	1.127	3.025E-01	5.234E-01
P56480	ATP SYNTHASE, H+ TRANSPORTING MITOCHONDRIAL F1 COMPLEX, BETA SUBUNIT	621	19	1.128	1.494E-05	2.832E-04

P57016	LADININ	10	2	1.129	1.217E-01	3.019E-01
Q9JLI6	SELENOCYSTEINE LYASE	6	1	1.129	2.945E-01	5.210E-01
Q99LX0	PARKINSON DISEASE (AUTOSOMAL RECESSIVE, EARLY ONSET) 7	35	3	1.133	1.134E-01	2.857E-01
P38060	3-HYDROXY-3-METHYLGLUTARYL-COENZYME A LYASE	8	2	1.133	4.830E-01	6.866E-01
Q9DCL9	PHOSPHORIBOSYLAMINOIMIDAZOLE CARBOXYLASE, PHOSPHORIBOSYLAMINORIBOSYLAMINOIMIDAZOLE, SUCCINOCARBOXAMIDE SYNTHETASE	5	2	1.133	1.611E-01	3.612E-01
P57746	ATPASE, H+ TRANSPORTING, LYSOSOMAL V1 SUBUNIT D	14	3	1.134	6.935E-01	8.377E-01
Q9R0Y5	ADENYLATE KINASE 1	7	1	1.139	2.521E-01	4.755E-01
P22315	FERROCHELATASE	8	3	1.140	4.602E-01	6.685E-01
P63158	HIGH MOBILITY GROUP BOX 1	17	4	1.141	2.374E-01	4.644E-01
Q9Z1Q5	CHLORIDE INTRACELLULAR CHANNEL 1	14	4	1.142	6.336E-01	8.044E-01
P67778	PROHIBITIN	13	4	1.142	4.258E-01	6.383E-01
Q9DB29	RIKEN CDNA 4833421E05 GENE	9	1	1.143	3.447E-01	5.658E-01
Q9DAK9	PHOSPHOHISTIDINE PHOSPHATASE 1	9	1	1.144	5.682E-01	7.589E-01
Q8QZS1	3-HYDROXYISOBUTYRYL-COENZYME A HYDROLASE	13	3	1.144	4.207E-01	6.354E-01
Q99KP3	CRYSTALLIN, LAMDA 1	40	4	1.144	2.073E-01	4.308E-01
P46471	PROTEASOME (PROSOME, MACROPAIN) 26S SUBUNIT, ATPASE 2	5	2	1.144	2.542E-01	4.773E-01
P52480	PYRUVATE KINASE, MUSCLE	55	9	1.144	3.512E-03	2.588E-02
Q01853	VALOSIN CONTAINING PROTEIN	40	12	1.145	2.290E-01	4.533E-01
Q9CQQ7	ATP SYNTHASE, H+ TRANSPORTING, MITOCHONDRIAL F0 COMPLEX, SUBUNIT B, ISOFORM 1	35	4	1.146	7.884E-02	2.249E-01
Q9WVL0	GLUTATHIONE TRANSFERASE ZETA 1 (MALEYLACETOACETATE ISOMERASE)	13	4	1.147	2.295E-01	4.532E-01
Q9CQX8	MITOCHONDRIAL RIBOSOMAL PROTEIN S36	11	3	1.147	1.611E-01	3.602E-01
Q04646	FXYD DOMAIN-CONTAINING ION TRANSPORT REGULATOR 2	7	1	1.147	3.708E-01	5.927E-01
Q8BJ64	RIKEN CDNA D630034H06 GENE	9	5	1.149	5.386E-01	7.341E-01
P99028	UBIQUINOL-CYTOCHROME C REDUCTASE HINGE PROTEIN	23	2	1.150	4.498E-01	6.569E-01
P99027	RIBOSOMAL PROTEIN, LARGE P2	14	2	1.151	1.129E-01	2.861E-01
Q60866	PHOSPHOTRIESTERASE RELATED	15	4	1.152	2.675E-01	4.906E-01
Q04857	PROCOLLAGEN, TYPE VI, ALPHA 1	15	4	1.152	5.960E-01	7.790E-01
P68372	TUBULIN, BETA 2C	31	4	1.152	2.288E-01	4.553E-01
P21107	TROPOMYOSIN 5	6	1	1.153	5.043E-01	7.031E-01
Q9CZR8	TS TRANSLATION ELONGATION FACTOR, MITOCHONDRIAL	7	2	1.154	2.431E-01	4.697E-01
P12658	CALBINDIN-28K	105	8	1.155	4.580E-03	3.143E-02
O70439	SYNTAXIN 7	5	1	1.155	2.865E-01	5.148E-01
Q8BP40	LYSOPHOSPHATIDIC ACID PHOSPHATASE	7	2	1.155	4.657E-01	6.716E-01
P16858	SIMILAR TO GLYCERALDEHYDE-3-PHOSPHATE DEHYDROGENASE	150	7	1.156	6.771E-04	6.823E-03
P21614	GROUP SPECIFIC COMPONENT	7	2	1.157	7.193E-02	2.136E-01
Q8VDN2	ATPASE, NA+/K+ TRANSPORTING, ALPHA 1 POLYPEPTIDE	317	20	1.157	1.391E-05	2.768E-04
P13707	GLYCEROL-3-PHOSPHATE DEHYDROGENASE 1 (SOLUBLE)	15	3	1.157	2.375E-01	4.634E-01
Q3THE2	MYOSIN LIGHT CHAIN, REGULATORY B	19	4	1.158	2.274E-01	4.549E-01

P14094	ATPASE, NA+/K+ TRANSPORTING, BETA 1 POLYPEPTIDE	40	3	1.159	6.479E-03	3.937E-02
O88343	ELECTROGENIC SODIUM BICARBONATE COTRANSPORTER 1	15	6	1.160	3.700E-01	5.926E-01
P17182	ENOLASE 1, ALPHA NON-NEURON	461	14	1.160	1.290E-06	3.422E-05
Q91WT8	CDNA SEQUENCE BC013481	10	2	1.160	1.395E-01	3.286E-01
Q9D6R2	ISOCITRATE DEHYDROGENASE 3 (NAD+) ALPHA	6	3	1.161	5.379E-01	7.344E-01
Q60605	MYOSIN, LIGHT POLYPEPTIDE 6, ALKALI, SMOOTH MUSCLE AND NON-MUSCLE	28	2	1.162	7.450E-02	2.180E-01
Q62433	N-MYC DOWNSTREAM REGULATED GENE 1	53	6	1.163	3.012E-02	1.175E-01
O08638	MYOSIN, HEAVY POLYPEPTIDE 11, SMOOTH MUSCLE	22	10	1.164	1.775E-01	3.809E-01
P57780	ACTININ ALPHA 4	58	15	1.164	6.703E-03	4.012E-02
P68368	TUBULIN, ALPHA 4	25	2	1.167	5.982E-02	1.890E-01
Q9R0P3	ESTERASE D/FORMYLGLUTATHIONE HYDROLASE	26	4	1.169	4.166E-02	1.461E-01
P25444	RIBOSOMAL PROTEIN S2	25	3	1.170	1.029E-01	2.722E-01
Q99NB1	ACYL-COA SYNTHETASE SHORT-CHAIN FAMILY MEMBER 1	47	6	1.174	1.129E-01	2.853E-01
Q62418	DREBRIN-LIKE	5	2	1.174	1.718E-03	1.440E-02
Q6ZQM8	UDP GLUCURONOSYLTRANSFERASE 1 FAMILY, POLYPEPTIDE A7C	9	2	1.175	4.007E-01	6.193E-01
Q91W43	DNA SEGMENT, CHR 19, WAYNE STATE UNIVERSITY 57, EXPRESSED	14	3	1.175	5.614E-01	7.549E-01
Q9CZU6	CITRATE SYNTHASE	16	3	1.177	2.090E-01	4.310E-01
Q9DCW4	RIKEN CDNA 0610009I16 GENE	109	9	1.180	1.389E-03	1.215E-02
P68037	UBIQUITIN-CONJUGATING ENZYME E2L 3	7	1	1.180	4.622E-01	6.689E-01
Q9Z2V4	PHOSPHOENOLPYRUVATE CARBOXYKINASE 1, CYTOSOLIC	156	13	1.181	1.491E-04	1.799E-03
Q9DCZ4	RIKEN CDNA 0610008C08 GENE	6	2	1.181	4.694E-01	6.720E-01
P47963	RIBOSOMAL PROTEIN L13	8	2	1.183	3.981E-01	6.189E-01
P11352	GLUTATHIONE PEROXIDASE 1	33	6	1.183	4.943E-02	1.639E-01
P18242	CATHEPSIN D	12	2	1.186	3.272E-02	1.252E-01
P05533	LYMPHOCYTE ANTIGEN 6 COMPLEX, LOCUS A	5	1	1.188	5.848E-01	7.745E-01
P10493	NIDOGEN 1	8	3	1.190	3.072E-01	5.270E-01
P17742	PEPTIDYLPROLYL ISOMERASE A	31	3	1.191	6.588E-02	2.001E-01
P42669	PURINE RICH ELEMENT BINDING PROTEIN A	13	3	1.194	1.396E-01	3.278E-01
Q58A65	SPERM ASSOCIATED ANTIGEN 9	6	2	1.196	1.765E-01	3.796E-01
Q9D0I9	RIKEN CDNA 2610011N19 GENE	8	2	1.198	3.371E-01	5.590E-01
P45952	ACETYL-COENZYME A DEHYDROGENASE, MEDIUM CHAIN	50	6	1.198	1.096E-02	5.555E-02
O35215	D-DOPACHROME TAUTOMERASE	8	2	1.200	1.325E-01	3.215E-01
Q9DCX2	ATP SYNTHASE, H+ TRANSPORTING, MITOCHONDRIAL F0 COMPLEX, SUBUNIT D	48	5	1.201	5.684E-03	3.591E-02
P62814	ATPASE, H+ TRANSPORTING, LYSOSOMAL V1 SUBUNIT B2	67	10	1.202	3.968E-04	4.327E-03
Q9D1K2	ATPASE, H+ TRANSPORTING, LYSOSOMAL V1 SUBUNIT F	6	1	1.205	3.468E-01	5.669E-01
Q99JY3	GTPASE, IMAP FAMILY MEMBER 4	5	1	1.206	6.258E-01	8.009E-01
P40142	TRANSKETOLASE	72	7	1.206	4.258E-03	2.947E-02
Q8CCK0	H2A HISTONE FAMILY, MEMBER Y3	36	1	1.207	6.367E-02	1.957E-01
Q99LC3	NADH DEHYDROGENASE (UBIQUINONE) 1 ALPHA SUBCOMPLEX 10	33	5	1.210	3.265E-02	1.255E-01
Q9WVE8	PROTEIN KINASE C AND CASEIN KINASE SUBSTRATE IN NEURONS 2	8	1	1.214	2.673E-01	4.914E-01
Q8BGA8	RIKEN CDNA C730027J19 GENE	34	8	1.215	2.297E-03	1.847E-02

P09671	SUPEROXIDE DISMUTASE 2, MITOCHONDRIAL	10	1	1.215	4.841E-01	6.844E-01
Q8CGP5	HISTONE 1, H2AF	90	2	1.216	3.059E-06	7.379E-05
Q9CYR0	SINGLE-STRANDED DNA BINDING PROTEIN 1	5	1	1.217	3.213E-01	5.384E-01
Q9CXZ1	NADH DEHYDROGENASE (UBIQUINONE) FE-S PROTEIN 4	11	2	1.217	1.537E-01	3.496E-01
Q99K51	PLASTIN 3 (T-ISOFORM)	5	1	1.219	3.069E-01	5.277E-01
P63028	TUMOR PROTEIN, TRANSLATIONALLY-CONTROLLED 1	9	1	1.224	1.092E-01	2.785E-01
O35387	HCLS1 ASSOCIATED X-1	7	1	1.229	2.268E-01	4.547E-01
Q9EQ20	ALDEHYDE DEHYDROGENASE FAMILY 6, SUBFAMILY A1	227	13	1.232	1.223E-10	5.122E-09
Q8R0F8	FUMARYLACETOACETATE HYDROLASE DOMAIN CONTAINING 1	76	4	1.232	1.280E-03	1.132E-02
Q8K3J1	NADH DEHYDROGENASE (UBIQUINONE) FE-S PROTEIN 8	8	1	1.235	3.695E-01	5.930E-01
Q99LB7	SARCOSINE DEHYDROGENASE	36	11	1.235	2.052E-02	9.025E-02
P42125	DODECENOYL-COENZYME A DELTA ISOMERASE (3,2 TRANS-ENOYL-COENYME A ISOMERASE)	32	5	1.236	7.728E-02	2.229E-01
P28352	APURINIC/APYRIMIDINIC ENDONUCLEASE 1	5	1	1.237	3.005E-01	5.223E-01
Q9Z1G3	ATPASE, H+ TRANSPORTING, LYSOSOMAL (VACUOLAR PROTON PUMP) 42KD	8	2	1.240	1.577E-01	3.565E-01
Q8K4Z3	APOA-I BINDING PROTEIN	8	2	1.241	4.725E-02	1.600E-01
Q9DBF1	DNA SEGMENT, CHR 18, WAYNE STATE UNIVERSITY 181, EXPRESSED	33	6	1.241	1.950E-01	4.097E-01
Q91XE0	GLYCINE-N-ACYLTRANSFERASE	16	4	1.243	1.412E-01	3.297E-01
P46460	N-ETHYLMALEIMIDE SENSITIVE FUSION PROTEIN	17	5	1.249	1.916E-02	8.520E-02
Q9WV27	ATPASE, NA+/K+ TRANSPORTING, ALPHA 4 POLYPEPTIDE	23	1	1.253	6.769E-02	2.041E-01
P61922	HYPOTHETICAL PROTEIN, I54	28	8	1.264	1.978E-02	8.748E-02
Q8K010	5-OXOPROLINASE (ATP-HYDROLYSING)	17	7	1.265	1.078E-02	5.538E-02
Q9CQA3	SUCCINATE DEHYDROGENASE COMPLEX, SUBUNIT B, IRON SULFUR (IP)	24	5	1.267	3.055E-02	1.180E-01
P46412	GLUTATHIONE PEROXIDASE 3	16	3	1.273	5.516E-02	1.785E-01
Q91WD5	EXPRESSED SEQUENCE AL033311	17	4	1.273	6.774E-02	2.035E-01
Q9CQC7	NADH DEHYDROGENASE (UBIQUINONE) 1 BETA SUBCOMPLEX 4	13	1	1.276	4.107E-01	6.299E-01
Q64522	SIMILAR TO HISTONE H2A	6	1	1.278	3.662E-03	2.674E-02
Q8CAY6	T-COMPLEX PROTEIN 1, RELATED SEQUENCE 1	17	4	1.279	1.688E-01	3.723E-01
Q91WS0	DNA SEGMENT, CHR 10, ERATO DOI 214, EXPRESSED	20	3	1.282	7.604E-02	2.209E-01
Q9DB77	UBIQUINOL CYTOCHROME C REDUCTASE CORE PROTEIN 2	60	7	1.285	3.203E-03	2.383E-02
P46935	NEURAL PRECURSOR CELL EXPRESSED, DEVELOPMENTALLY DOWN-REGULTED GENE 4	10	3	1.286	5.517E-01	7.494E-01
Q78ZA7	NUCLEOSOME ASSEMBLY PROTEIN 1-LIKE 4	5	2	1.286	5.316E-02	1.741E-01
Q9JHW2	NITRILASE FAMILY, MEMBER 2	9	2	1.287	2.204E-02	9.485E-02
Q8C5H8	RIKEN CDNA 1110020G09 GENE	9	4	1.288	4.623E-04	4.907E-03
P04117	FATTY ACID BINDING PROTEIN 4, ADIPOCYTE	11	1	1.289	3.052E-02	1.185E-01
Q99JY0	HYDROXYACYL-COENZYME A DEHYDROGENASE/3-KETOACYL-COENZYME A THIOLASE/ENOYL-COENZYME A HYDRATASE (TRIFUNCTIONAL PROTEIN), BETA SUBUNIT	15	5	1.290	1.426E-01	3.309E-01
Q8VDW0	DEAD (ASP-GLU-ALA-ASP) BOX POLYPEPTIDE 39	5	2	1.295	1.220E-01	3.016E-01
Q9ERS2	NADH DEHYDROGENASE (UBIQUINONE) 1 ALPHA SUBCOMPLEX, 13	10	2	1.301	1.012E-02	5.335E-02
Q8K009	ALDEHYDE DEHYDROGENASE 1 FAMILY, MEMBER L2	7	1	1.304	2.512E-01	4.760E-01

Q9D855	UBIQUINOL-CYTOCHROME C REDUCTASE BINDING PROTEIN	28	5	1.305	1.027E-01	2.726E-01
Q9R0Q7	PROSTAGLANDIN E SYNTHASE 3 (CYTOSOLIC)	12	1	1.307	5.106E-01	7.069E-01
P48771	CYTOCHROME C OXIDASE, SUBUNIT VIIA 2	11	1	1.310	1.142E-01	2.858E-01
P10649	GLUTATHIONE S-TRANSFERASE, MU 1	32	4	1.310	1.198E-04	1.490E-03
Q91YT0	NADH DEHYDROGENASE (UBIQUINONE) FLAVOPROTEIN 1	27	6	1.311	1.325E-01	3.225E-01
P47757	CAPPING PROTEIN (ACTIN FILAMENT) MUSCLE Z-LINE, BETA	8	1	1.313	9.834E-02	2.681E-01
P52503	NADH DEHYDROGENASE (UBIQUINONE) FE-S PROTEIN 6	8	2	1.317	9.249E-02	2.530E-01
P97816	S100 CALCIUM BINDING PROTEIN G	240	3	1.318	3.994E-19	7.948E-17
Q71RI9	CYSTEINE CONJUGATE-BETA LYASE 2	7	1	1.330	5.348E-05	7.884E-04
Q8R2Y8	RIKEN CDNA A230072I16 GENE	6	2	1.330	4.665E-01	6.715E-01
P13020	GELSOLIN	8	4	1.330	3.211E-01	5.393E-01
Q9D2G2	DIHYDROLIPOAMIDE S-SUCCINYLTRANSFERASE (E2 COMPONENT OF 2-OXO-GLUTARATE COMPLEX)	47	2	1.336	2.984E-03	2.262E-02
Q9WTP6	ADENYLATE KINASE 2	50	8	1.348	3.686E-03	2.667E-02
Q9CZY3	UBIQUITIN-CONJUGATING ENZYME E2 VARIANT 1	7	2	1.350	1.881E-01	3.982E-01
Q8BK30	RIKEN CDNA 1500032D16 GENE	15	1	1.350	2.973E-02	1.171E-01
Q99K67	AMINOADIPATE-SEMIALDEHYDE SYNTHASE	64	15	1.350	4.938E-03	3.303E-02
Q91Y97	ALDOLASE 2, B ISOFORM	171	13	1.351	2.434E-07	7.177E-06
P05201	GLUTAMATE OXALOACETATE TRANSAMINASE 1, SOLUBLE	19	2	1.354	5.612E-02	1.801E-01
Q99KI0	ACONITASE 2, MITOCHONDRIAL	238	21	1.358	3.442E-15	3.044E-13
Q9D819	PYROPHOSPHATASE (INORGANIC) 1	7	2	1.360	1.510E-01	3.463E-01
Q9CQH0	PDZK1 INTERACTING PROTEIN 1	11	1	1.360	2.339E-02	9.905E-02
Q9CQJ8	NADH DEHYDROGENASE (UBIQUINONE) 1 BETA SUBCOMPLEX, 9	5	2	1.363	1.853E-01	3.943E-01
Q9WUR9	ADENYLATE KINASE 3 ALPHA-LIKE 1	14	4	1.366	3.464E-02	1.295E-01
P52760	HEAT-RESPONSIVE PROTEIN 12	42	5	1.369	3.800E-06	8.897E-05
Q921H8	ACETYL-COENZYME A ACYLTRANSFERASE 1A	50	9	1.370	3.044E-03	2.286E-02
Q9DBJ1	PHOSPHOGLYCERATE MUTASE 1	117	6	1.370	4.261E-08	1.413E-06
Q9DCM2	GLUTATHIONE S-TRANSFERASE KAPPA 1	7	1	1.373	3.543E-02	1.306E-01
Q9D0S9	HISTIDINE TRIAD NUCLEOTIDE BINDING PROTEIN 2	168	2	1.378	4.936E-18	6.548E-16
Q925I1	ATPASE FAMILY, AAA DOMAIN CONTAINING 3A	6	2	1.379	5.581E-02	1.799E-01
Q9DCM0	ETHYLMALONIC ENCEPHALOPATHY 1	27	3	1.380	2.139E-03	1.737E-02
P52825	CARNITINE PALMITOYLTRANSFERASE 2	16	4	1.388	9.944E-05	1.319E-03
P70296	PHOSPHATIDYLETHANOLAMINE BINDING PROTEIN 1	30	4	1.392	3.933E-05	6.261E-04
Q9CPU0	RIKEN CDNA 1110008E19 GENE	15	4	1.397	9.953E-03	5.282E-02
P19536	CYTOCHROME C OXIDASE, SUBUNIT VB	11	2	1.412	3.374E-02	1.273E-01
P50544	ACYL-COENZYME A DEHYDROGENASE, VERY LONG CHAIN	33	10	1.415	8.571E-03	4.805E-02
P63242	EUKARYOTIC TRANSLATION INITIATION FACTOR 5A	66	3	1.417	2.542E-05	4.215E-04
P26443	GLUTAMATE DEHYDROGENASE 1	91	11	1.419	7.385E-10	2.799E-08
Q9D3D9	ATP SYNTHASE, H+ TRANSPORTING, MITOCHONDRIAL F1 COMPLEX, DELTA SUBUNIT	18	1	1.419	1.584E-02	7.332E-02
Q8R3P0	ASPARTOACYLASE (AMINOACYLASE) 2	10	2	1.427	1.365E-02	6.584E-02
Q9QYR9	ACYL-COA THIOESTERASE 2	18	2	1.442	5.164E-03	3.369E-02

O35409	FOLATE HYDROLASE	14	4	1.442	7.631E-02	2.209E-01
Q9QZQ8	H2A HISTONE FAMILY, MEMBER Y	12	2	1.444	5.082E-03	3.343E-02
Q61656	DEAD (ASP-GLU-ALA-ASP) BOX POLYPEPTIDE 5	5	2	1.459	2.708E-03	2.134E-02
Q61838	PREGNANCY ZONE PROTEIN	5	1	1.487	1.687E-01	3.731E-01
P29391	FERRITIN LIGHT CHAIN 1	27	4	1.507	3.321E-02	1.265E-01
Q9CQ75	NADH DEHYDROGENASE (UBIQUINONE) 1 ALPHA SUBCOMPLEX, 2	6	1	1.532	1.391E-01	3.295E-01
Q00519	XANTHINE DEHYDROGENASE	8	4	1.532	2.361E-02	9.890E-02
O55137	**ACYL-COA THIOESTERASE 1**	**14**	**6**	**1.547**	**5.044E-03**	**3.346E-02**
Q8BXK9	RIKEN CDNA 5730531E12 GENE	10	3	1.558	4.578E-02	1.571E-01
P09803	CADHERIN 1	8	1	1.578	3.658E-02	1.336E-01
Q9DB20	**ATP SYNTHASE, H+ TRANSPORTING, MITOCHONDRIAL F1 COMPLEX, O SUBUNIT**	**13**	**2**	**1.590**	**1.711E-03**	**1.449E-02**
Q8BFZ3	**RIKEN CDNA 4732495G21 GENE**	**8**	**1**	**1.599**	**7.420E-03**	**4.343E-02**
Q9QXS1	PLECTIN 1	5	4	1.619	5.059E-01	7.040E-01
O09111	NADH-UBIQUINONE OXIDOREDUCTASE ESSS SUBUNIT, MITOCHONDRIAL PRECURSOR	11	1	1.636	2.240E-02	9.533E-02
P62204	**CALMODULIN 1**	**17**	**2**	**1.639**	**4.259E-05**	**6.520E-04**
P10605	**CATHEPSIN B**	**23**	**3**	**1.647**	**1.622E-05**	**3.002E-04**
Q9D6J5	NADH DEHYDROGENASE (UBIQUINONE) 1 BETA SUBCOMPLEX 8	9	1	1.653	2.123E-01	4.323E-01
Q8K183	**PYRIDOXAL (PYRIDOXINE, VITAMIN B6) KINASE**	**8**	**2**	**1.657**	**7.216E-04**	**7.180E-03**
Q3UFF7	LYSOPHOSPHOLIPASE-LIKE 1	7	2	1.658	7.511E-02	2.190E-01
Q61033	THYMOPOIETIN	5	3	1.663	3.102E-01	5.299E-01
P08905	LYSOZYME	6	1	1.686	1.011E-01	2.700E-01
Q9D892	**INOSINE TRIPHOSPHATASE (NUCLEOSIDE TRIPHOSPHATE PYROPHOSPHATASE)**	**9**	**1**	**1.700**	**2.110E-03**	**1.732E-02**
P24472	**GLUTATHIONE S-TRANSFERASE, ALPHA 4**	**7**	**1**	**1.712**	**9.167E-04**	**8.898E-03**
Q9D8N0	**EUKARYOTIC TRANSLATION ELONGATION FACTOR 1 GAMMA**	**14**	**2**	**1.721**	**2.179E-06**	**5.596E-05**
Q9WU79	**PROLINE DEHYDROGENASE**	**13**	**4**	**1.729**	**1.553E-04**	**1.845E-03**
Q9D8I3	**RIKEN CDNA 2010001H14 GENE**	**6**	**1**	**1.756**	**1.010E-04**	**1.318E-03**
Q68FL4	**HYPOTHETICAL PROTEIN, MNCB-5555**	**11**	**1**	**1.777**	**9.237E-04**	**8.858E-03**
Q9JHL1	**SOLUTE CARRIER FAMILY 9 (SODIUM/HYDROGEN EXCHANGER), ISOFORM 3 REGULATOR 2**	**11**	**1**	**1.783**	**3.625E-04**	**4.007E-03**
Q80UU9	PROGESTERONE RECEPTOR MEMBRANE COMPONENT 2	6	1	1.792	1.662E-02	7.605E-02
O35887	CALUMENIN	5	2	1.809	3.492E-02	1.299E-01
Q8QZW3	**CDNA SEQUENCE BC026682**	**6**	**1**	**1.832**	**9.024E-03**	**4.920E-02**
O09131	GLUTATHIONE S-TRANSFERASE OMEGA 1	5	1	1.851	2.033E-01	4.237E-01
Q9R257	HEME BINDING PROTEIN 1	6	1	1.877	2.187E-02	9.460E-02
P10518	AMINOLEVULINATE, DELTA-, DEHYDRATASE	14	1	1.957	7.334E-02	2.162E-01
Q9D1L0	**COILED-COIL-HELIX-COILED-COIL-HELIX DOMAIN CONTAINING 2**	**5**	**1**	**1.966**	**2.950E-03**	**2.258E-02**
Q8BWT1	**ACETYL-COENZYME A ACYLTRANSFERASE 2 (MITOCHONDRIAL 3-OXOACYL-COENZYME A THIOLASE)**	**344**	**12**	**2.012**	**2.787E-54**	**2.218E-51**
P41216	**ACYL-COA SYNTHETASE LONG-CHAIN FAMILY MEMBER 1**	**9**	**5**	**2.060**	**4.729E-03**	**3.217E-02**
Q8R3F5	**CDNA SEQUENCE BC025519**	**5**	**1**	**2.089**	**1.111E-03**	**1.017E-02**

Q60770	SYNTAXIN BINDING PROTEIN 3A	5	3	2.100	3.936E-01	6.168E-01
Q8VCW8	**CDNA SEQUENCE BC018371**	**16**	**2**	**2.251**	**2.038E-05**	**3.687E-04**
Q9CQ60	**6-PHOSPHOGLUCONOLACTONASE**	**6**	**2**	**2.275**	**8.295E-03**	**4.716E-02**
Q9CZ83	MITOCHONDRIAL RIBOSOMAL PROTEIN L55	5	1	2.310	6.553E-02	1.999E-01
P09528	**FERRITIN HEAVY CHAIN 1**	**22**	**5**	**2.319**	**6.153E-07**	**1.689E-05**
Q9CXS4	**PROLINE-RICH POLYPEPTIDE 6**	**5**	**1**	**2.689**	**1.135E-04**	**1.457E-03**
P13745	**GLUTATHIONE S-TRANSFERASE, ALPHA 1 (YA)**	**19**	**2**	**2.697**	**9.608E-05**	**1.296E-03**
Q7TMF3	NADH DEHYDROGENASE (UBIQUINONE) 1 ALPHA SUBCOMPLEX, 12	6	1	2.850	1.127E-02	5.679E-02
P24549	**ALDEHYDE DEHYDROGENASE FAMILY 1, SUBFAMILY A1**	**25**	**5**	**2.991**	**5.311E-04**	**5.420E-03**
O88587	CATECHOL-O-METHYLTRANSFERASE	7	2	3.017	5.428E-02	1.771E-01
P97434	**EXPRESSED SEQUENCE AA536749**	**9**	**2**	**4.421**	**9.594E-04**	**9.092E-03**
P04228	**HISTOCOMPATIBILITY 2, CLASS II ANTIGEN A, ALPHA**	**8**	**1**	**6.435**	**1.751E-03**	**1.452E-02**
